# Rice Husks as a Biogenic Template for the Synthesis of Fe_2_O_3_/MCM-41 Nanomaterials for Polluted Water Remediation

**DOI:** 10.3390/molecules30122484

**Published:** 2025-06-06

**Authors:** Tamara B. Benzaquén, Paola M. Carraro, Griselda A. Eimer, Julio Urzúa-Ahumada, Po S. Poon, Juan Matos

**Affiliations:** 1 Centro de Investigación y Tecnología Química (CITeQ), UTN-CONICET, Maestro Marcelo López esq. Cruz Roja Argentina, Córdoba 5016ZAA, Argentina; 2 Proyecto ANILLO: Efficient Use of Water for Sustainable Agriculture Under Climate Change Condition (H_2_O-SAC^3^), Universidad Autónoma de Chile, Santiago 8900000, Chile; urzua.jaer@gmail.com; 3 Unidad de Desarrollo Tecnológico (UDT), Universidad de Concepción, Barrio Universitario s/n, Concepción 4070386, Chile; 4 Unidad de Cambio Climático y Medio Ambiente (UCCMA), Instituto Iberoamericano de Desarrollo Sostenible (IIDS), Facultad de Arquitectura, Construcción y Medio Ambiente, Universidad Autónoma de Chile, Temuco 4780000, Chile

**Keywords:** biogenic SiO_2_, rice husks, MCM-41 supports, Fe-based photocatalysts, rhodamine B

## Abstract

This work shows a sustainable methodology for the synthesis of biogenic materials designed for the removal and photodegradation of rhodamine B (RhB), a highly dangerous environmental pollutant that induces reproductive toxicity. The classical synthesis of MCM-41-ordered mesoporous materials was modified using biocompatible rice husk as the silica template. Iron was incorporated and the so-prepared biogenic photocatalysts were characterized by X-ray diffraction, N_2_ adsorption–desorption isotherms, transmission electron microscopy, diffuse reflectance UV-Vis, surface pH, cyclic voltammetry, and Fourier transform infrared spectral analysis of pyridine adsorption. The photocatalytic performance of the materials was evaluated following the removal by adsorption and the photon-driven degradation of RhB. The adsorption capacity and photocatalytic activity of the biogenic materials were correlated with their properties, including iron content, texture, surface content, and electrochemical properties. The best biogenic material boosted the degradation rates of RhB under UV irradiation up to 4.7 and 2.2 times greater than the direct photolysis and the benchmark semiconductor TiO_2_-P25. It can be concluded that the use of rice husks for the synthesis of biogenic Fe-modified mesoporous materials is a promising strategy for wastewater treatment applications, particularly in the removal of highly toxic organic dyes.

## 1. Introduction

The rapid development of industrialization and modernization processes causes severe effects on the environment. Among them, the worldwide concern regarding water pollution has grown in recent years. Textile industries are responsible for producing the greatest amount of liquid effluent because of the extensive use of water in the dyeing process [1]. Currently, over 9000 distinct synthetic dyes are utilized, each corresponding to a specific class of chemical applications in a range of products, such as clothing, food, and leather goods. Approximately 10–15% of the unused dyes are released as waste [2]. Specifically, the release of dyes into water bodies alters their coloration, decreases oxygen levels, and inhibits the growth of aquatic plants. [3,4]. Furthermore, some dyes have been identified as carcinogenic and non-biodegradable [2]. Environmental scientists are currently faced with the crucial task of effectively eliminating these contaminants from water. Several physical and chemical technologies have been developed to face this problem, but they are not always environmentally friendly, low-cost, or highly effective [5,6,7,8]. In addition, most of them are not fully selective processes, since they depend on the type and nature of the effluents. In addition, although biological treatment processes may be a suitable choice, issues such as toxicity, low biodegradability, and seasonal production can affect the treatment efficacy.

Moreover, with these treatments it is not possible to achieve the legal limits of discharge for effluents with the presence of textile organic dyes. In this way, some specific chemical processes, known as advanced oxidation processes (AOPs) [9,10,11,12,13,14,15,16,17,18,19,20,21,22,23,24], have been shown to be efficient alternatives for the degradation of organic molecules, including dyes [16,17,18,19,20,21,22] and microplastics [23,24]. A common characteristic of AOPs, including ozonation [13,14], ultrasonication [15,16], gamma radiation [17], hydrogen peroxide [18], Fenton [19], photo-Fenton [20], photocatalysis [21,22], and electro photocatalysis [23,24], is the production of highly reactive oxygen species (ROS) such as hydroxy (•OH) and superoxo (O_2_•^−^) radicals [13,22,25]. ROS are responsible for driving oxidation processes yielding degradation and the total mineralization of pollutants.

We have reported [21,22] several biogenic materials playing the role of microreactors, largely increasing the accessibility of pollutants from the aqueous phase to the reactive active sites. Innovative and cost-effective functional materials can be developed to prepare ordered mesoporous materials with uniform pore sizes.

The increasing interest for the application of these materials in environmental treatment is due to the high capacity for the adsorption of pollutants in the aqueous phase, which is determined by the high surface area and specific sites permitting the selective removal of pollutants [26,27]. For instance, ordered mesoporous materials such as MCM-41 are of significant interest due to their superior pore architecture and high specific surface area. These properties are particularly effective for the degradation of pollutants, as they enable optimal dispersion of the active catalytic phase and facilitate the diffusion of larger molecules to the active sites located within the mesopores.

However, the main silica precursors used in the synthesis of these materials are alkoxysilanes, such as tetramethoxysilane (TMOS) and tetraethoxysilane (TEOS), which are considered harmful for human health. Thus, in the search for a safer, less costly, and more environmentally friendly precursor, natural silica—commonly called biogenic silica—can provide a low-cost alternative source to replace commercial silica precursors. Biogenic silica can be found in agricultural residues, such as wheat straw [28,29] and rice husks [30,31,32,33], one of the most important agricultural crops, with a global production of 600 million tons per year, producing more than 100 million tons of husks per year [34].

On the other hand, it is well-known that TiO_2_ is the benchmark photocatalyst under UV irradiation [35,36]. However, TiO_2_ exhibits operative limitations [37], including low photon absorption in the visible range and low surface area. In addition, it has been reported that TiO_2_ is a suspected human carcinogen [38]. Therefore, photocatalysts based on metal oxides other than TiO_2_ are required to be used in the solar-driven remediation of polluted water.

Thus, it is necessary to use alternative, low-cost, stable under high-photon flux conditions, and biocompatible photocatalysts. In this sense, we have reported that nanoporous carbon-supported Fe-based photocatalysts showed remarkable heterogeneous Fenton-like photoactivity [22]. In this sense, our group has early reported the photocatalytic degradation of rhodamine B (RhB) on a biogenic photocatalyst containing amorphous carbon and rice husks-derived silica [21].

Thus, it is of high interest to verify this behavior using ordered mesoporous materials such as MCM silica. Accordingly, the main objective of this work is to verify the photocatalytic activity of biogenic mesoporous molecular sieves prepared from rice husks as a natural, non-toxic, and cheap source of silica modified with iron loadings. RhB was selected as a target model pollutant to verify the photoactivity of biogenic silica supports, and the results were compared to both the ordered mesoporous silica obtained using a traditional method and the benchmark TiO_2_-P25.

## 2. Results and Discussion

### 2.1. Characterization of Catalysts

#### 2.1.1. Iron Content, Structural and Textural Properties

MCM-41 and MCM-RHA materials were prepared according to the synthesis route described in Section 3.1. Then, these SiO_2_-based supports were subsequently modified with different nominal iron loadings with respect to MCM-41 and MCM-RHA, and the materials were identified as Fe/MCM-41(x) and Fe/MCM-RHA(x), where x symbolizes the nominal Fe content (2.5, 5, or 10% *w*/*w*).

**Figure 1:**
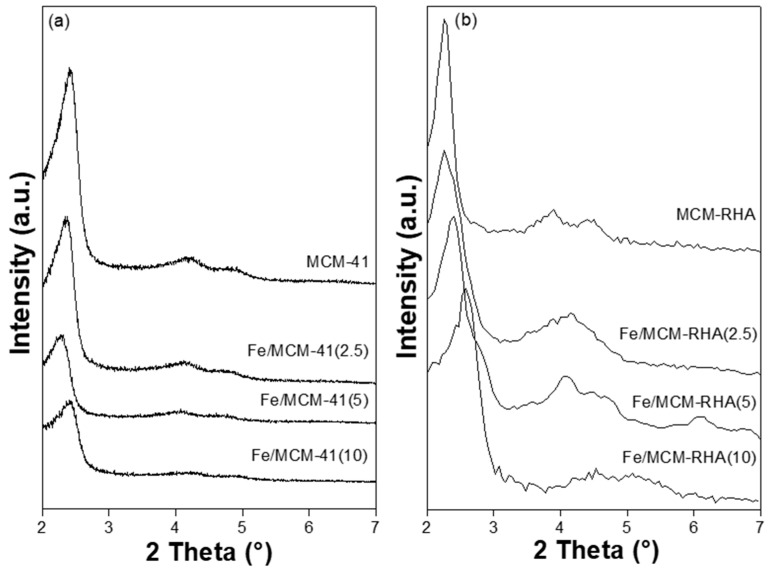
XRD patterns of supports and catalysts. (**a**) MCM-41 series; (**b**) MCM-RHA series.

Figure 1 shows the X-ray diffraction patters (XRD) of MCM-41, MCM-RHA, and the two series of Fe-based catalysts. The samples exhibit an XRD pattern typical of the MCM-41 mesoporous material, with a main peak located at the small angle of ca. 2.5°. The intensity of this peak monotonically decreases with the increase in Fe loading, suggesting the formation of Fe-based clusters [39]. The MCM-41 series of catalysts presents a main intense peak at low-angle reflection between 2.3–2.4°, corresponding to the distance between (100) planes, while the other two weak peaks at ca. 4.2° and 4.8° were attributed to reflections of (110) and (200) planes, typical of the MCM-41 mesoporous structure [40]. On the other hand, despite the fact that the MCM-RHA series has the same main peak at a low-angle observed between 2.3–2.6°, it can be also seen that the minor intense peaks have shifted to higher diffraction angles. Furthermore, it is clear that the samples with more Fe content have shifted, and the peak became broader compared to the MCM-41 series, which is indicative of an important change in the chemical environment of the MCM structure. All diffraction data were recorded with the same scan rate of 0.6°·min−1. Thus, the remarkable changes observed between Fe-containing MCM-41- and MCM-RHA-supported catalysts can be attributed to the small differences in the crystalline framework interpreted in terms of some metallic impurities presented in the rice husks-derived silica, such as Al and Cu detected by EDS analysis for the MCM-RHA series, according to Figure S1 (SI). It can also be seen that not only is the intensity of the peaks in the MCM-RHA series higher than the MCM-41 series, but it is also more amorphous than the MCM-41 materials. This is inferred from the increase in the noise of the XRD patterns. No characteristic diffraction’s peaks of iron oxide were identified in the low-angle XRD patterns (Figure 1). Thus, Fe oxide species would be in an amorphous state or would be clusters/particles with a very small size (below the XRD detection limit). This fact has already been observed by us elsewhere [39,40].

**Figure 2:**
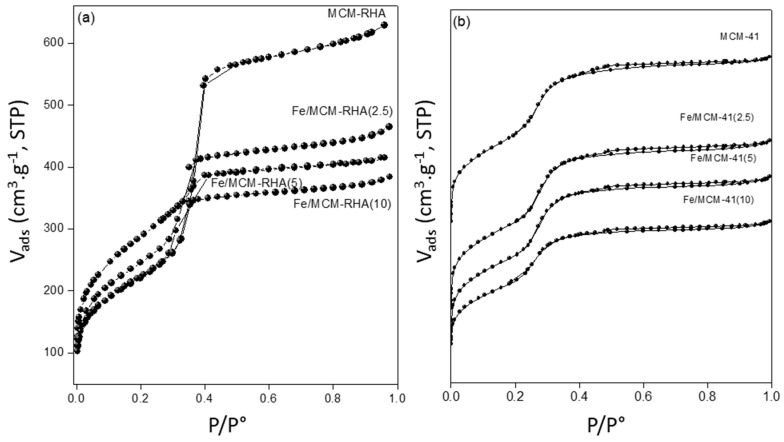
N_2_ adsorption/desorption isotherms of supports and catalysts. (**a**) MCM-RHA series; (**b**) MCM-41 series.

The mesoporous framework of the samples was confirmed by N_2_ adsorption–desorption isotherms [39], as shown in the Figure 2. As can be seen, the isotherms followed a type IV that is typical of mesopore materials, with an H4 hysteresis in the desorption branch. The iron content detected from the ICP analysis and the textural properties obtained from the N_2_ adsorption–desorption isotherms for the two series of catalysts are compiled in Table 1. The iron content detected by the ICP analysis is close to the nominal values expected, and more importantly, the catalysts had similar Fe content among both series, permitting a proper comparison of the photocatalytic results discussed below.

All of the samples presented average pore diameters, pore volume, and S_BET_ areas that are typical of mesoporous materials. However, several features are highlighted. First, the increase in Fe content monotonically decreases the specific surface area (S_BET_) in both series of catalysts. The decrease in the surface area of the MCM supports, as a consequence of the incorporation of a metallic active phase, has been previously observed by our group in the case of mesoporous pillared carbon supports [41]. The formation of small particles of Fe-based species is commonly located in the micropore framework of the supports; however, as discussed above, the micropore volume of the present MCM series of materials is negligible, and accordingly, it is expected that Fe-based particles formed clusters in the mesopore framework, which are responsible for the decrease in the specific surface area of MCM-41 and MCM-RHA supports.

For instance, the influence of Fe content on textural properties is more important in the MCM-RHA than that observed in the MCM-41 series. For instance, MCM-41 decreases S_BET_ by 5%, 7%, and 20% after the incorporation of 2.5 wt. %, 5 wt. %, and 10 wt.% iron content, respectively. In contrast, MCM-RHA decreases the S_BET_ by 13%, 14%, and 26% after the incorporation of 2.5 wt. %, 5 wt. %, and 10 wt.% iron content, respectively. Furthermore, Table 1 shows that the effect of iron content is much more pronounced on the total volume of pores (V_TP_). This property suffered a remarkable decrease of 19%, 27%, and 32% after it incorporated 2.5 wt. %, 5 wt. %, and 10 wt.% of iron content in MCM-RHA, respectively, while V_TP_ only decreased by 16% in Fe/MCM-41(10) compared to pure MCM-41. This result confirms that the formation of iron oxides clusters blocking the mesopore framework of the silica, as suggested by the decrease in the mean pore diameter observed in the MCM-RHA series. Regarding the meso- and microporosity, it should be noted that the mesoporous volume is generated by the removal of the template used. Since the total volume quantifies both micropores and mesopores, the present materials do not have micropores. This was confirmed by the α-plot method, which showed that the micropore volume is zero [39,42]. Therefore, the total pore volume is equal to the mesopore volume.

In addition, it seems to be clear that the chemical properties of silica-based support, such as the surface pH, influence the dispersion of iron oxide-based species on the surface of the catalytic supports, as discussed below in Section 2.1.3.

#### 2.1.2. Transmission Electron Microscopy (TEM) and UV-Visible Absorption Spectroscopy (UV-Vis/DR)

**Figure 3:**
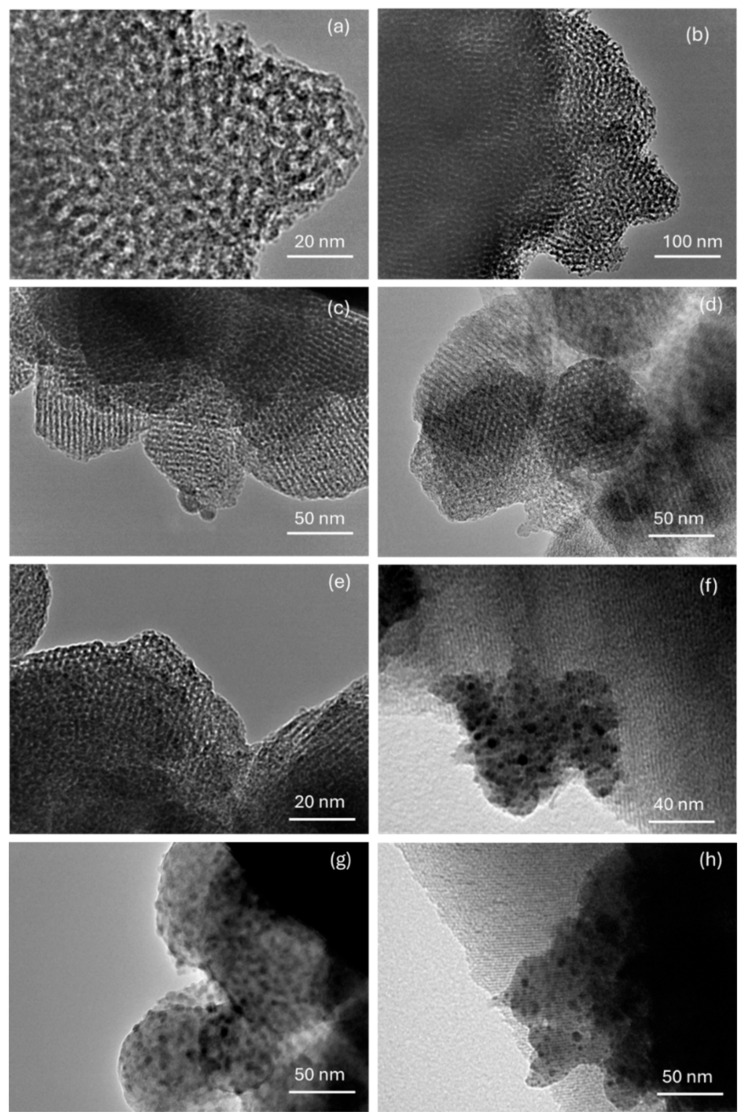
Composition of transmission electron microscopy (TEM) images of supports and catalysts. (**a**) MCM-41; (**b**) MCM-RHA; (**c**) Fe/MCM-41(2.5); (**d**) Fe/MCM-RHA(2.5); (**e**) Fe/MCM-41(5); (**f**) Fe/MCM-RHA(5); (**g**) Fe/MCM-41(10); (**h**) Fe/MCM-RHA(10).

The morphology and some structural features of the materials were examined by transmission electron microscopy (TEM), as shown in Figure 3. Figure 3a and Figure 3b show the TEM images of the MCM-41 and MCM-RHA supports, respectively. It can be seen that most of the particles seem to be constituted by pores with a spherical-like morphology, and with a diameter range between 80–160 nm. Similar inferences have been reported by Liu and coworkers [43] on the TEM study of Pt nanowires produced within mesoporous MCM-41, and by Ibrahim and coworkers [44] on their study on sulfated Zr supported in the MCM-41 composite.

Nowadays, although the present samples have a highly ordered mesoporous structure, the particles with Fe-based catalysts do not have a well-defined morphology. In addition, there is an aggregation of nanoparticles of irregular sizes. For instance, the TEM images in Figure 3c,d show that the catalysts with lower Fe content, Fe/MCM-41(2.5) and Fe/MCM-RHA(2.5), respectively, are more ordered and have regular diameter sizes, compared to samples with higher Fe content (Figure 3e–h). However, in any of the cases, all of the catalysts show a well-defined mesoporous structure where both the frontal and longitudinal views of mesopores are observed.

The porous framework observed in Figure 3 agrees with the structural regularity inferred from XRD patterns and N_2_ adsorption–desorption isotherms reported elsewhere [39,42]. In addition, the presence of Fe-containing nanoparticles embedded within the mesoporous channels of the silica supports can be inferred from the dark spots observed in Figure 3f–h. These particles showed a range of sizes between 3 and 6 nm (with a range of sizes about 3–6 nm), which can be responsible for the decrease observed in the specific surface area and the total volume of pores by the blocking of mesopores discussed above.

Unfortunately, we do not have TEM-EDS, but we consider that there is no doubt that the dark spots observed in Figure 3 correspond to Fe nanoparticles, as confirmed by SEM-EDS in Figure S1 in the Supplementary Information (SI) for the sample Fe/MCM-RHA(10). It should be highlighted that SEM-EDS is a surface analysis technique and, therefore, according to the low apparent weight content of Fe observed for this sample, is further evidence that most of the Fe nanoparticles are confined within the SiO_2_-based mesoporous framework, which agrees with the decrease in the specific surface area discussed above.

It is clear that XRD and SEM-EDS analysis demonstrated the presence of Fe-based nanoparticles within the mesoporous framework of the SiO_2_-based materials. On the other hand, in previous works [39,42], UV-Vis spectra was employed to verify the optical properties of iron-based species incorporated within MCM-41 and MCM-RHA mesoporous materials. Figure S2 (SI) shows the UV-Visible spectra for Fe-based catalysts. It is interesting to highlight that the intensity in the absorbance band is more pronounced for the sample with the highest Fe content. In addition, a deeper analysis permits us to confirm that the materials exhibit three main absorption regions. A first band located between 230–300 nm has been assigned to the dπ–pπ charge transfer between Fe and O, suggesting that unsaturated Fe^3+^ ions linked to O atoms of the silica-based supports, which can induce strong Lewis acidity [45] in the material, as discussed below in Section 2.1.3. A second absorption band observed between 300 and 400 nm has been ascribed to the charge transfer process from O^2−^ to Fe^3+^ in small-sized Fe_2_O_3_ species [40]. This charge transfer process is responsible for the band gap commonly observed in the UV region [46]. For instance, Matchim-Fondjo et al. [47] demonstrated that the optical band gap obtained from UV–Vis gradually decreases from 3.09 eV to 2.52 eV with the increases of iron content [48] supported on ZnO.

Moreover, the presence of different Fe species can be inferred from the UV-Vis, where the characteristic bands of the spectrum correspond to Fe^3+^ in Fe_2_O_3_ species, with different sizes and interactions with the matrix. The increase in intensity and the shifting to the visible range as iron loading increased has been ascribed to a quantum size effect [49]. The position of the absorption edge of semiconductor powders is strongly influenced by particle size, which shifts significantly toward the lower wavelength by decreasing the particle size. Consequently, the shape and the size of FeO oxides affected the UV region absorption, causing changes in the shape and position. Therefore, the shifting of the UV band position observed suggests that the FeO nanoclusters or nanoparticles predominant in the samples with higher Fe loadings should be larger, and should probably have a weaker interaction with the matrix [50]. A third absorption peak, centered at ca. 500 nm, corresponds to relatively larger iron oxide nanoparticles [47,51], probably on the surface of the silica, which agrees with the nanoparticles observed in Figure 3d. In summary, the UV-Vis of the catalysts shows the presence of iron oxide clusters/nanoparticles increasing with Fe content, which could be responsible for the blocking of some mesopores. This inference agrees with the loss of the specific surface area and total volume of pores discussed above. The sample with the lower Fe loading is present in the higher proportion of isolated Fe^3+^ ions linked to O atoms of the silica.

In addition, for the sake of comparison, Figure S3 (SI) shows the XPS spectra in the Fe 2p core level for the Fe/MCM-RHA(10) catalyst. The spectra can be deconvoluted into four peaks assigned to the Fe^3+^ chemical state. According to the literature [40], the binding energy of Fe^3+^ 2p^3/2^ at 711.5 eV, accompanied by a satellite line visible at 720 eV, and the binding energy of Fe^3+^ 2p^1/2^ at 725 eV, are indicative of the presence of Fe^3+^ in the oxide environment. In addition, these peaks are broader compared to the narrow lines of bulk oxides [40], which can be considered as evidence of a high dispersion of metal species on the silica.

#### 2.1.3. Chemisorption of Pyridine and Surface pH

**Figure 4:**
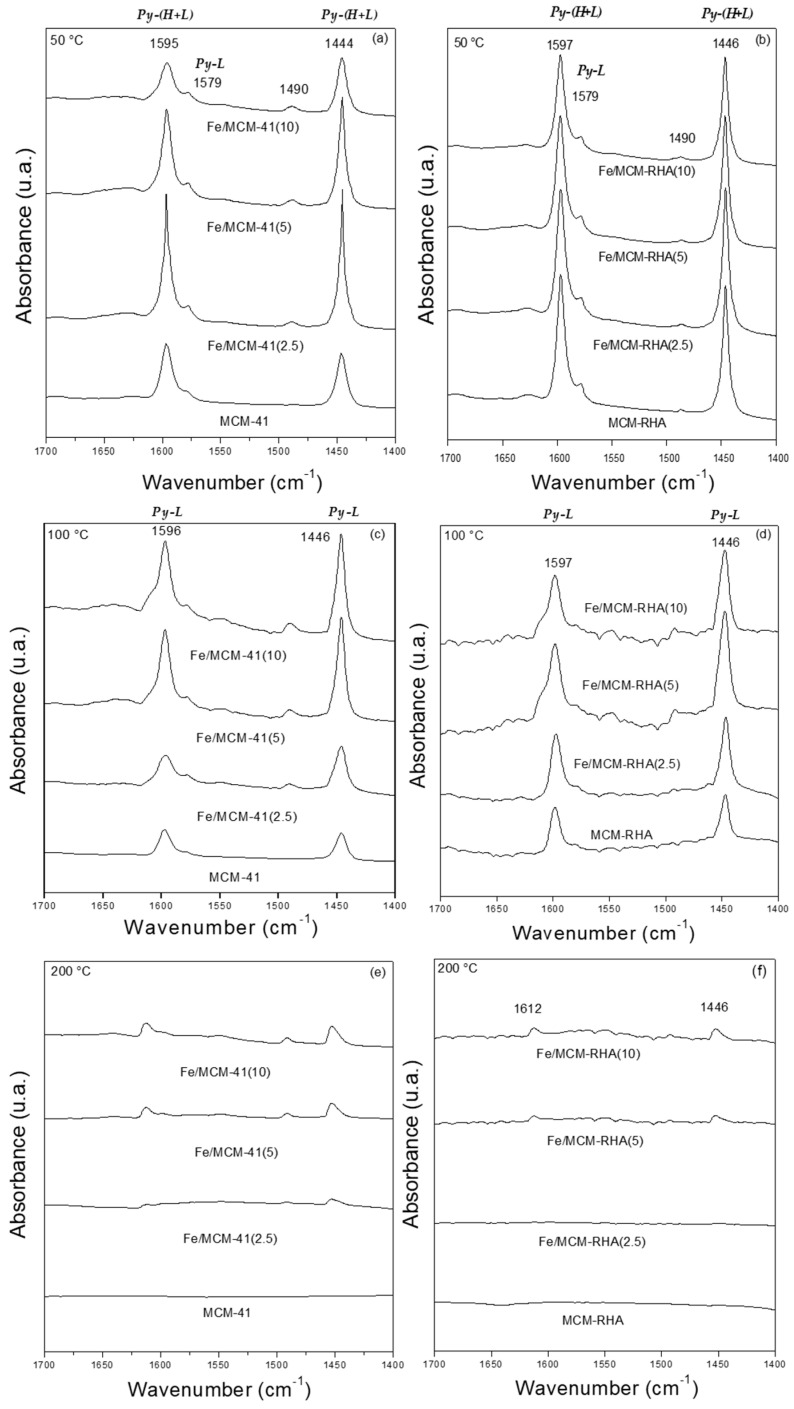
FTIR spectra of pyridine chemisorption after desorption step. (**a**,**b**) 50 °C; (**c**,**d**) 100 °C; (**e**,**f**) 200 °C.

The chemisorption of pyridine followed by FT-IR is used to identify Lewis and Brönsted acidic sites on solid catalysts. Accordingly, this characterization technique provides a better understanding of the nature of different species present in the samples. Figure 4 shows that the IR spectra referred to pyridine chemisorption after the thermo-desorption process at 50, 100, and 200 °C. Pyridine is a basic molecule with a p*K*_b_ of ca. 8.77 [52] that can interact with acid sites via the nitrogen lone-par electrons giving rise to different characteristic bands. According to the classical acid–base concepts, p*K_b_* is equivalent to the negative logarithm of base dissociation constant (p*K_b_* = −log *K_b_*), and, therefore, it is a chemical descriptor used to measure the basic strength of a molecule. Thus, a low p*K_b_* is associated with a strong base. Accordingly, the desorption at different temperatures provides information about the strength of the Lewis and Brönsted acid sites of the materials. For instance, pyridine may form hydrogen bonds (Py-H) with the silanol (SiOH) groups of the silica supports. The bands observed at 1446 and 1596 cm^−1^ for the MCM-41 and MCM-RHA supports correspond to hydrogen-bonded pyridine [53], and these are the only bands found in the present samples. Interestingly, these bands disappeared upon desorption at 200 °C (Figure 4e,f), indicating a weak interaction between pyridine and SiOH groups.

Additionally, adsorbed pyridine forming Lewis-type adducts could be identified by IR adsorption bands at about 1600–1620 and 1445–1455 cm^−1^. Therefore, the bands at 1446 and 1596 cm^−1^ in the samples modified with iron could be interpreted in terms of the overlapping of both the hydrogen-bonded pyridine band and a band associated with pyridine that is coordinately bonded to Lewis’s acid sites (Lewis-type adduct). These bands are still present, even after desorption at 200 °C, for the samples with higher Fe loadings, indicating their acidic strength. In addition, the shift to a higher wavelength is observed, evidencing the presence of other types of acid sites. Thus, the intensity of these bands in the Fe-modified samples increases with the Fe loading, which is further evidence of the overlapping of both the hydrogen-bonded pyridine band (Py-H) and a band attributed to pyridine that is coordinately bonded to Lewis’s acid sites (Py-L), (Figure 4e,f).

In addition, it is possible to observe in Figure 4b,d, compared to Figure 4a,c, signals with a higher intensity, which could indicate a higher number of acid sites in MCM-RHA materials. The Lewis acidity can be attributed to the presence of electron-deficient Fe^3+^ species interacting with the probe molecule through its electron pair; this interaction could arise from the isolated Fe species coordinated with the framework of silica through oxygen atoms (Fe-O-Si bonds). Such a kind of Fe-doped silica can be present in the structure of oxide nanoclusters or nanoparticles in a similar shape to that reported by Carraro and coworkers for the Zn/MCM-41 catalyst [54].

On the other hand, bands corresponding to Brønsted acid sites at ~1540 cm^−1^ and 1636 cm^−1^ have not been detected. However, Figure 4a–d show the presence of a small band at ca. 1580 cm^−1^, which can be attributed to pyridine adsorbed in Lewis’s acid sites [55,56]. Therefore, the band at 1490 cm^−1^, which is frequently associated with pyridine and Lewis and Brönsted acid sites, could only be assigned to Lewis’s acid sites, which clearly increase with the amount of Fe in the material [53].

**Figure 5:**
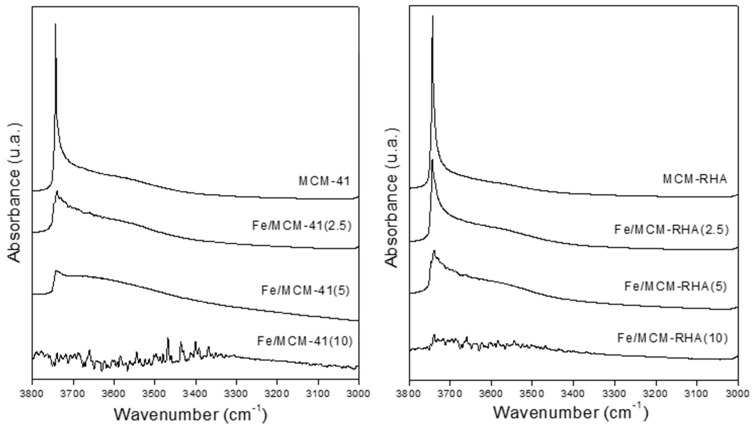
FTIR spectra in the hydroxyl stretching region after degassing at 400 °C.

Figure 5 shows the IR spectra in the hydroxyl range before pyridine adsorption, followed by desorption at 400 °C. The samples containing the lowest iron loadings and their respective supports showed an intense band at 3740 cm^−1^, corresponding to the stretching vibrations of isolated hydroxyl groups. However, this band decreases or disappears with increasing Fe loading, which could be attributed to the condensation of some Si–OH groups with iron species, leading to the formation of Si–O–Fe bonds [40] and contributing to the Lewis acidity.

**Figure 6:**
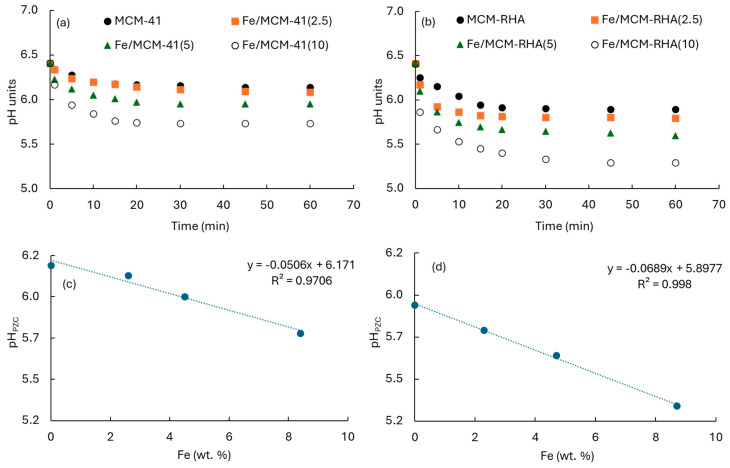
(**a**,**b**) pHs of catalysts as a function of time. (**c**,**d**) pH_PZC_ as a function of experimental Fe content. (**c**) MCM-41; (**d**) MCM-RHA.

Concerning the surface pH analysis, Figure 6a,b show the evolution of pH as a function of time for the MCM-41 and MCM-RHA series of catalysts, respectively, which are suspended in the aqueous phase. After 30 min, pH achieved a steady-state condition of equilibrium, suggesting the surface of the material is characterized by a zero charge. These pH values correspond to the point-zero charge pH (pH_PZC_) summarized in Table 1. The pH_PZC_ decreases when Fe content increases, as can be seen in Figure 6c,d for the MCM-41 and MCM-RGA series of catalysts, respectively. A linear relationship between pH_PZC_ and Fe content can be seen. This is a common fact in both series, but changes are more marked in the MCM-RHA series (curve with steepest slope). For instance, the pH_PZC_ decreases after 60 min from 6.14 for MCM-41 to 6.08, 5.95, and 5.73 after the incorporation of ca. 2.6 wt. %, 4.5 wt. %, and 8.4 wt.% iron. However, the changes are clearly higher for the MCM-RHA series, decreasing from 5.89 to 5.74, 5.59, and 5.29 after the incorporation of ca. 2.3 wt. %, 4.7 wt. %, and 8.7 wt.% iron.

These trends agree with the pyridine chemisorption tests discussed above, demonstrating that the biogenic silica-based mesoporous material led to a more acidic surface compared to that of the MCM-41 series, and therefore, it may interact selectively with basic molecules in the aqueous phase. Accordingly, differences in both adsorption and photocatalytic degradation of rhodamine B are expected to be found, as discussed in Section 3.2 and Section 3.3.

#### 2.1.4. Electrochemical Characterization

**Figure 7:**
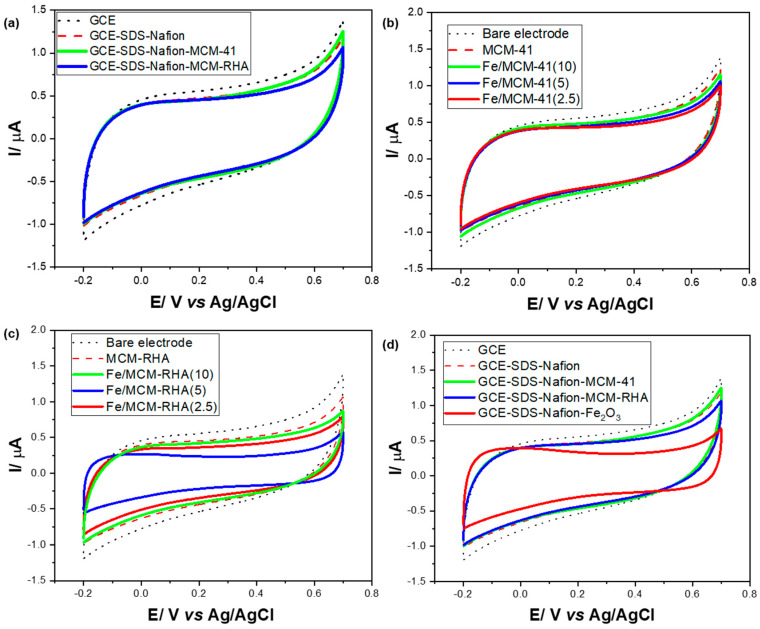
Electrochemical cells with 10 mL of 0.1 M PBS. (**a**) CVs of bare GCEs and modified GCEs with SDS-Nafion and the silica-based materials in the absence of Fe; (**b**,**c**) CVs of GCEs modified with Fe/MCM-41 and Fe/MCM-RHA catalysts. (**d**) CVs of GCE and modified GCE with MCM-41, MCM-RHA series, and homemade Fe_2_O_3_.

For a correct interpretation of the electrochemical results, Figure 7a shows the cyclic voltammetry (CV) study for bare MCM-41 and MCM-RHA in the absence of iron. For the sake of comparison, the response of unmodified glassy carbon electrodes (GCEs) is also shown, along with electrodes modified with the dispersant and adhesive (SDS and Nafion).

The CVs of the electrodes modified with the MCM-41 and MCM-RHA are very similar to those obtained from the unmodified electrodes. However, a slight decrease in the currents can be observed when the GCEs are modified with MCM-41 and MCM-RHA. This result indicates a decrease in the electroactive area on the surface of the electrodes due to the high electric isolator behavior of SiO_2_ [55]. No oxidation-reduction signal (Faradaic current) was detected in any electrode; only capacitive currents are observed, probably as a result of the ions’ organization of the electrolyte on the surface of the electrodes. Figure 7b shows the CVs obtained from GCEs modified for the Fe/MCM-41 series of catalysts. The capacitive currents of the modified electrodes decrease compared to the bare GCE, and when the Fe content decreases, the capacitive currents of the modified GCEs with MCM-41 also decrease. This fact is more evident in the MCM-RHA series, as can be seen in Figure 7c. However, unlike the MCM-41 series, the decrease in the capacitive current is not proportionally related to the Fe content in the samples, showing the following order in the capacitive current of Fe/MCM-RHA(10) > Fe/MCM-RHA(2.5) > Fe/MCM-RHA(5).

On the other hand, it is important to highlight that the modified GCEs with any of the two Fe-containing series of catalysts did not show the oxidation-reduction signals commonly attributed to the redox pair Fe^3+^/Fe^2+^ [56] or even Fe^2+^/Fe^0^ [57]. The voltammograms observed so far do not reflect any type of redox process attributed to iron, at least in the potential window used in the present work.

The decrease in the electroactive area observed in all of the electrodes modified with the samples, together with the absence of faradaic oxidation-reduction signals, suggest that the modifier compounds act as silica-based insulators [55], where Fe species were apparently not susceptible to gain or lose electrons throughout the volume of material deposited on the electrodes. To verify this inference, Figure 7d shows a GCE modified with a homemade, prepared Fe_2_O_3_, and an even greater decrease in capacitive currents was observed.

The reduction signal for the Fe^3+^ to Fe^+2^, commonly attributed [55] to the process of Fe_2_O_3_ + 4H^+^ + 2e^−^ → 2 FeOH^+^ + H_2_O (E° = 0.16 V vs. SHE), was not observed, and this could be due to the near-neutral pH of the electrolyte. Table 2 shows a summary of the voltammogram area (μA·V^−1^) and the electrical charge (μC) obtained on the present MCM-41 and MCM-RHA series of catalysts at a scan rate of 0.05 V·s^−1^. For the sake of comparison, the values obtained on GCE and homemade Fe_2_O_3_ are also included. The electrical charge was calculated by dividing the voltammogram area (μA·V^−1^) by the sweep speed applied (0.05 V·s^−1^). Compared to GCE modified only with the dispersant (SDS-Nafion), the voltammogram area for the electrodes modified with MCM-41 and MCM-RHA decreased by a factor of only ca. 1% and 5%, respectively. However, once the MCM-41 is modified with iron, after an immediate reduction of the voltammogram area after the incorporation of ca. 2.5 wt.% Fe, this parameter increases at ca. 5 wt.% Fe content and becomes higher that that observed on MCM-41 when 10 wt. % iron was embedded in the silica-based support. In contrast, when iron is incorporated into MCM-RHA, the voltammogram area remarkably decreased to ca. 0.444 (μA·V^−1^) for the GCE modified with Fe/MCM-RHA(10), and in spite of this, the parameter suffered an increase at 10 wt.% Fe added, and this value was still lower than that observed on the GCE modified with MCM-RHA. In other words, while Fe content added to MCM-41 samples seems to slightly compensate for the drop in the capacitive current, this trend is not observed in the Fe-modified MCM-RHA samples.

### 2.2. Kinetics of Adsorption in the Dark

**Figure 8:**
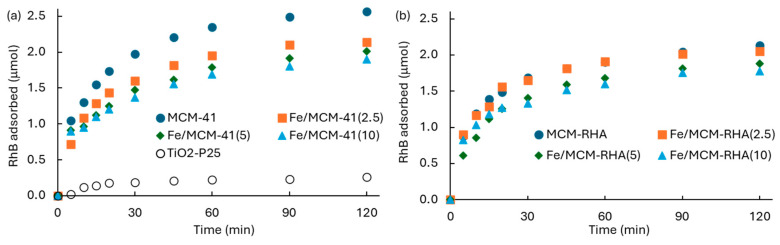
Kinetics of RhB adsorption in the dark on Fe-based MCM-based photocatalysts. (**a**) MCM-41; (**b**) MCM-RHA.

Figure 8 shows the kinetics of RhB adsorption (q_t_) on the different Fe-containing MCM-based photocatalysts. The influence of Fe content on the MCM-41 and MCM-RHA adsorption capacity is shown in Figure 8a,b, respectively. For the sake of comparison, Figure 8a also contains the adsorption of RhB on the semiconductor benchmark of TiO_2_-P25.

All of the supports and catalysts show the equilibrium condition for the RhB adsorption at ca. 60 min, suggesting there is a strong affinity of the materials for the adsorption of this pollutant. Table 3 contains a summary of the RhB adsorbed at an equilibrium condition after 60 min, and the other kinetic parameters discussed below. An important decrease in the RhB adsorption of both the MCM-41 and MCM-RHA series of catalysts as a function of the iron content can be seen. For instance, the removal of RhB after 60 min (steady-state condition of equilibrium) decreases from 2.35 μmol for MCM-41 to 1.96, 1.79, and 1.69 μmol for Fe/MCM-41(2.5), Fe/MCM-41(5), and Fe/MCM-41(10), respectively. This means there is a decrease in the RhB adsorption of ca. 17%, 24%, and 28% for Fe/MCM-41(2.5), Fe/MCM-41(5), and Fe/MCM-41(10), respectively, relative to MCM-41.

This behavior can be firstly attributed to a decrease in the surface area of support due to the incorporation of iron. It is expected that the pore size distribution of the materials suffered changes as a consequence of the pore being blocking by Fe-based agglomerated species, leading to a reduction in the total active sites for the adsorption of the pollutant. Although the average diameter of pores remains constant in the MCM-41 series of catalysts, it suffered a reduction from 3.0 nm to 2.6 nm in the MCM-RHA series, which is consistent with the lower RhB adsorption values shown by these materials. This fact has also been reported for the adsorption of Lu^3+^ on modified MCM-41 support [56] and on biodiesel production using MoO_3_/MCM-41 catalysts [57]. In addition, the decrease in the adsorption capacity of MCM supports can be attributed to changes in the surface pH observed with the increase in iron content (Table 1). It is well-known that RhB became a positive zwitterion in the aqueous phase [21,58,59], and therefore its adsorption is not favored on acid surfaces. The decrease in the RhB removal is not so markedly in the MCM-RHA series. For instance, both MCM-RHA and Fe/MCM-RHA(2.5) adsorbed similar RhB amounts after 60 min (ca. 1.91 μmol). In addition, despite the fact that the surface pHs of the Fe-based MCM-RHA catalysts are clearly lower than the analogous series of MCMC-41 catalysts, after the incorporation of higher amounts of Fe, the RhB removal decreased to 1.68 and 1.60 μmol, which results in a reduction of only ca. 12% and 16% for Fe/MCM-RHA(5) and Fe/MCM-RHA(10), respectively, relative to MCM-RHA. However, a deep observation of data in Table 1 indicates that after the incorporation of 2.5 wt. % of Fe, the specific surface area and point-zero charge pH of MCM-41 only decreased 5% and 1%, respectively, while the reduction observed for both variables on MCM-RHA are ca. 13% and 2.5%, respectively. This reduction is clearly lower than those in the MCM-41 series, suggesting a combination of both surface area and pH are the driving force for the removal of the pollutant in the aqueous phase. Accordingly, for a better understanding of RhB adsorption on the Fe-modified MCM silica catalysts, the pseudo first-order [22,60,61,62,63], pseudo second-order [22,60], and intraparticle diffusion (IPD) [22,61,62,63] models were applied to the adsorption kinetic data using Equation (1), Equation (2), and Equation (3), respectively.

log(q_eq_ − q_t_) = log(q_t_) − (k_1_/2.303)·t (1)

[1/(q_eq_ − q_t_)] = (1/q_eq_) + k_2_·t (2)

q_t_ = C_IPD_ + k_IPD_·t^1/2^ (3)

**Figure 9:**
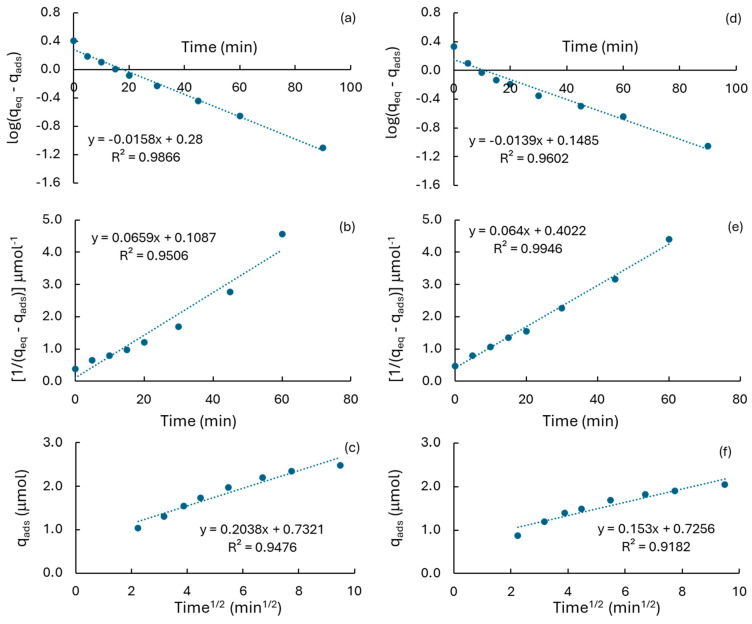
Mathematical treatment of RhB adsorption data for different kinetic models. (**a**–**c**) MCM-41; (**d**–**f**) MCM-RHA; (**a**,**d**) first-order kinetics; (**b**,**e**) second-order kinetics; (**c**,**f**) intraparticle diffusion (IPD).

Table S1 in the Supplementary Information (SI) shows a summary of the kinetic models used for the analysis of the kinetic data of RhB adsorption, where q_t_ and q_eq_ are the amount of RhB adsorbed (μmol) at time t (min) and at the equilibrium condition, respectively. The plots of log(q_eq_ − q_t_) and [1/(q_eq_ − q_t_)], as a function of time, according to Equation (1) and Equation (2), respectively, yield the pseudo first-order (k_1_, min^−1^) and the pseudo second-order rate-constants (k_2,_ μmol^−1^.min^−1^) for the adsorption of RhB. The kinetic order can be associated with the physisorption and chemisorption of pollutants in the aqueous phase [23,64]. The plot of q_t_ = f(t^1/2^), according to Equation (3), permits the estimation of the intraparticle diffusion (IPD) rate-constant (k_IPD_, μmol·min^−1/2^), and the capacity constant (C_IPD_, μmol) attributed to the extension of the boundary layer thickness [61,62,63] of molecules close to the surface of adsorbents when the time of process is close to zero. Thus, keeping in mind the differences observed in the pyridine chemisorption and the surface pH of silica-based supports, Figure 9 shows the plots of RhB adsorption on MCM-41 and MCM-RHA in terms of the three different kinetic models. Figures S4–S6 (SI) show the plots obtained on the Fe-based series of catalysts as a function of the iron content, and Figure S7 (SI) shows the plots for the RhB adsorption on TiO_2_-P25. The values of q_eq_, k_1_, k_2_, k_IPD_, and C_IPD_, and the linear regression factors (R^2^_k1_, R^2^_k2_, and R^2^_IPD_) for each kinetic model, are listed in Table 3. It is interesting to remark on the fact that k_1_ and k_2_ are practically similar for the adsorption of RhB on MCM-41 and MCM-RHA. In contrast, k_IPD_ is clearly higher in MCM-41 than in MCM-RHA, suggesting that limitations in the diffusion of RhB from the bulk of the solution to the surface of MCM-RHA may be taking place, probably induced by the electrostatic repulsion between the more acidic surface of MCM-RHA and the positive zwitterion form of RhB.

In addition, the linear regression factor observed for the second-order kinetic model (R^2^_k2_) is close to the unit (0.995, Table 3) on MCM-RHA, while the value observed is far from the unit on MCM-41 (0.951, Table 3). Thus, the R^2^_k2_ values suggested that RhB may be preferentially chemisorbed on MCM-RHA, while RhB would be mainly physisorbed on MCM-41, as suggested by the higher value observed for R^2^_k1_ (0.987 against 0.960, Table 3). This is more evident in the benchmark of TiO_2_-P25, where the close-to-unit value for R^2^_k2_ suggests that chemisorption is the most favored mechanism of adsorption of RhB on this semiconductor. The analysis of data in Table 3 suggests that iron incorporation into the silica-based supports is affective in a similar way to the adsorption rate-constants. For instance, it can be seen that when Fe content is the lowest, both k_1_ and k_2_ increased, mainly in the Fe/MCM-RHA(2.5) catalyst. However, when iron content increases to ca. 5% and 10%, the kinetic constants decreased in proportions between 25–34%. It seems that the presence of both reduced and iron oxide-based nanoparticles inhibit the adsorption of the pollutant. This inference is reinforced by the fact that the rate-constant, according to the IPD model (k_IPD_), decreased in most of the catalysts, relative to the neat MCM supports. The boundary layer thickness constant for the IPD model (C_IPD_) also decreased in most of the catalysts, indicating that an important inhibition in the diffusion of RhB molecules from the bulk of solution to the surface of catalysts is taking place. C_IPD_ is a measure of the adsorbed molecules near the interface between the bulk of solution and the solid [61,62,63], serving as an indicator of the electrostatic affinity of RhB molecules for adsorption. The decrease in C_IPD_ at higher amounts of iron in the catalysts corroborates the reduced proximity of molecules to the catalyst surface, the diminished adsorption efficiency, and the weakened interaction between RhB molecules and the catalyst surface, as can also be seen in the almost one order magnitude value of C_IPD_ that is observed for TiO_2_, characterized by an acid surface pH of ca. 6.4 [21]. At the same time, chemisorption is the preferred mechanism for the adsorption of RhB, which is inferred from the close-to-unit values observed in the linear regression factors for the second-order kinetics when iron has been incorporated into MCM supports.

### 2.3. Photocatalytic Tests

**Figure 10:**
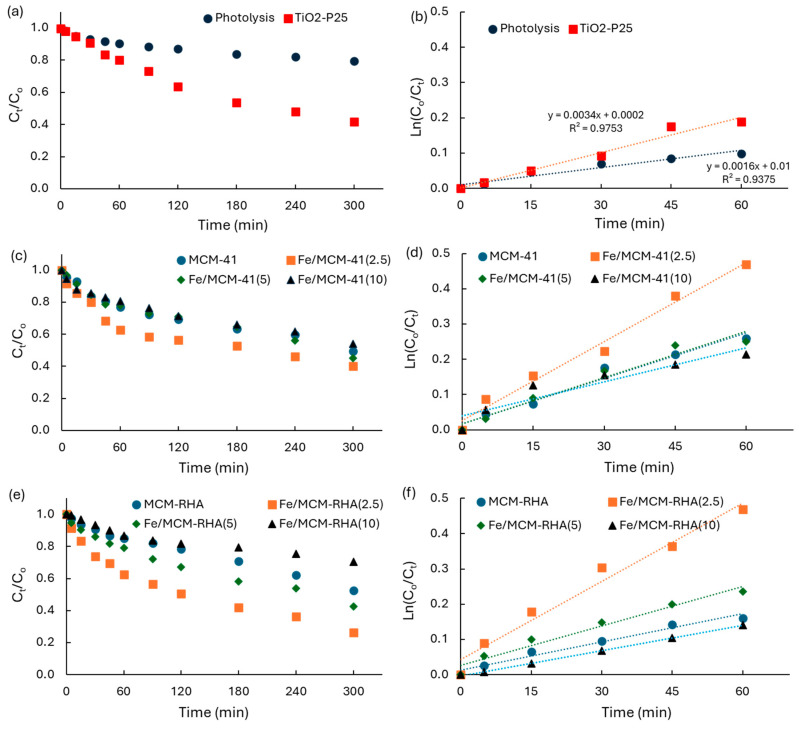
(**a**,**c**,**e**) Kinetics of RhB disappearance as a function of UV irradiation time; (**b**,**d**,**f**) linear regression of kinetic data according to a first-order mechanism of reaction.

According to results obtained from the kinetics of adsorption in the dark, 60 min is considered prior irradiation in the immersion photoreactor. This procedure permits a more accurate interpretation of the photocatalytic activity of the materials. Figure 10 shows the kinetics of RhB disappearance as a function of irradiation time, as well as the linear regression according to a first-order mechanism of the reaction.

Figure 10a shows the results for direct photolysis in the absence of solids, and the results using the benchmark of TiO_2_-P25. Table 4 shows a summary of the kinetic parameters obtained for the RhB photodegradation, including the first-order apparent rate-constants (k_app_, min^−1^) obtained from the linear regression of the kinetic data as a function of time obtained for the plot of Equation (4), and the photoconversion after 5 h of irradiation (C_5h_).

Ln(C_o_/C_t_) = k_app_·t (4)

Table 4 shows the photocatalytic activity of Fe-based catalysts supported on MCM-41 and MCM-RHA, relative to values obtained on direct photolysis (k_app-i_/k_lysis_), and relative to that using TiO_2_ (k_app-i_/k_TiO2_). As expected, due to the use of a high-power UV-lamp, the direct photolysis of RhB in the absence of catalysts is not negligible with a k_app_ of ca. 1.6 × 10^−3^ min^−1^ and a conversion of the ca. 20% of the pollutant after 5 h of UV irradiation. However, this activity is clearly lower than the benchmark semiconductor, with values of ca. k_app_ of ca. 3.4 × 10^−3^ min^−1^, and a ca. 58% conversion of the pollutant after 5 h of UV irradiation. Thus, the photoefficiency of TiO_2_-P25 is ca. 2.1 times higher than that observed on direct photolysis.

Table 4 contains some interesting results that merit being highlighted. MCM-41 and MCM-RHA are photocatalytically active in the absence of iron, with values of ca. 4.3 × 10^−3^ min^−1^ and 2.7 × 10^−3^ min^−1^, respectively, for the k_app_ which are up to 2.7 and 1.7 higher than the direct photolysis.

Furthermore, the photocatalytic activity of MCM-41 is 1.3 higher than that of TiO_2_-P25. This is a remarkable result for state-of-the art advanced oxidation technologies. However, the conversion of the pollutant after 5 h of UV irradiation is ca. 51% and 47% for MCM-41 and MCM-RHA, respectively, which is smaller than that observed on TiO_2_, indicating that the photocatalysts are deactivating alongside the reaction.

It should be mentioned that, given that the silica supports are composed solely of silicon and oxygen, they should not exhibit chemical activity. For this reason, various synthesis techniques are employed to incorporate active functions and endow these solids with activity. However, the photoactivity observed in the MCM-41 and MCM-RHA samples could be attributed to the adsorption of the contaminant onto these supports. This adsorption would favor its photolysis, thanks to the increased exposure and dispersion of contaminant molecules over the extensive surface area of the silica-based catalysts.

The incorporation of iron on MCM-41 and MCM-RHA yields an increase in the photocatalytic activity, with k_app_ values of ca. 7.5 × 10^−3^ min^−1^ and 7.4 × 10^−3^ min^−1^ for Fe/MCM-41(2.5) and Fe/MCM-RHA(2.5), respectively. These values represent an increase in the photocatalytic activity that is 4.7 and 2.2 times higher than that observed on direct photolysis and TiO_2_-P25, respectively, with a RhB conversion of up to 74% after 5h of irradiation on Fe/MCM-RHA(2.5). However, the increase in iron content up to 10% is clearly detrimental, reducing k_app_ to values even lower than those observed with the neat MCM supports. This detrimental effect is clearer for the MCM-RHA Fe-based catalysts, which can be due to the important reduction in the specific surface area due to the agglomeration of Fe-based species, and due to the more acidic surface of the MCM-RHA series of catalysts. To verify this inference, the global surface reaction (v_sur_, μmol.min^−1^) was estimated using Equation (5), where q_eq_ is the RhB adsorbed at an equilibrium condition after 60 min prior to UV irradiation (Table 3).

v_sur_ = q_eq_·k_app_ (5)

Our group used this equation early on [22,64] to normalize the photoactivity of different photocatalysts with a similar surface area. However, it should be mentioned that a comparison between MCM-based catalysts and TiO_2_ is not recommend in the present case, since the surface area of the benchmark semiconductor is clearly lower than that of the Fe-modified silica-based catalysts. Accordingly, the RhB adsorbed in the dark on TiO_2_ is much lower than the values adsorbed on the Fe/MCM-41 and Fe/MCM-RHA catalysts. For instance, v_sur_ is ca. 13.5 and 6.9 times higher on MCM-41 and MCM-RHA than TiO_2_-P25, but the conversion of RhB after 5 h of irradiation is slightly lower in the MCM materials than in TiO_2_.

However, v_sur_ values obtained using Equation (5) in terms of the RhB adsorbed in the dark at steady-state conditions (q_eq_) are clearly higher for all of the MCM-41 series of Fe-based catalysts than those on MCM-RHA catalysts. Thus, the use of Equation (5) is highly valuable to compare the photoactivity of Fe-containing catalysts against values obtained for the MCM supports in the absence of iron. According to these values, the Fe/MCM-RHA series of catalysts are more active than those supported on MCM-41. For instance, the comparison of v_sur_ for Fe/MCM-41(2.5) and Fe/MCM-RHA(2.5) against v_sur_ on MCM-41 and MCM-RHA yields values of 1.5 and 2.7, respectively. The higher photoactivity observed on the Fe/MCM-RHA series of catalysts, in terms of v_sur_, which also considers the q_eq_, is more coherent with 74% of RhB converted on Fe/MCM-RHA(2.5) instead of 60% for Fe/MCM-41(2.5).

### 2.4. General Discussion and Correlations

As discussed above, both series of catalysts exhibited similar iron (Fe) content among them, allowing for a reliable comparison of the results obtained. However, some differences in the adsorption and photocatalytic degradation of rhodamine B were found and merit being noted.

All materials showed average pore diameters, pore volumes, and specific surface areas (S_BET_), typical of mesoporous materials, and an increase in Fe content led to a monotonic decrease in S_BET_, with a more pronounced effect observed in the MCM-RHA series compared to the MCM-41 series. The surface pH of the silica-based support was affected by the dispersion of iron nano species. The point of zero charge decreased with increasing Fe content, with a linear relationship observed in both series, particularly in MCM-RHA, where a higher slope was appreciated. This was consistent with pyridine chemisorption tests, indicating a more acidic surface for the biogenic silica-based material. Moreover, these trends agree with the adsorption RhB results, which show that the adsorption capacity of the supports is strongly correlated with the observed changes in surface pH, and it was ascertained that RhB adsorption was not favored in the MCM-RHA series. This behavior could also be asserted due to additional limitations encountered in the diffusion of RhB from the bulk of the solution to the surface of MCM-RHA that may be taking place, probably induced by the electrostatic repulsion between the more acidic surface of MCM-RHA and the positive zwitterion form of RhB. Moreover, the kinetic parameters obtained for the RhB adsorption values suggested that RhB may be preferentially chemisorbed on MCM-RHA, while RhB would be mainly physisorbed on MCM-41. In addition, for both series of catalysts the presence of iron oxide-based species inhibit the adsorption of the pollutant, probably due to steric hindrance.

The photocatalytic results indicated that the effect of the distribution of iron species was comparable for both supports, where the samples containing a nominal metal loading of 2.5 wt.% exhibited the highest catalytic activity. As mentioned, the Fe samples with 2.5 wt.% contained the largest proportion of isolated cations, and the other samples demonstrated an increase in the overall concentration of metal species as the iron loading increased [42]. The stabilization of iron species as isolated cations is maintained depending on the level of metal loading until the saturation coverage of the support surface is achieved. As the Fe loading continues to rise, there is also an increase in the presence of Fe oxide clusters and/or nanoparticles of varying sizes, which may obstruct some active species and certain mesopores. On the other hand, although the MCM-RHA series can selectively interact with basic molecules in the aqueous phase, RhB removal was more pronounced in this series than in MCM-41. This could be because the more acidic surface of the MCM-RHA materials is associated with a greater number of Lewis-acidic isolated sites (isolated Fe^3+^) which, in turn, have been shown to have greater photocatalytic activity. Thus, in concordance with our previous studies [42], it is possible to reassert that the major photocatalytic efficiency is consistent with the highest presence of iron species (isolated Fe^3+^ ions) finely dispersed and stabilized on the silica structure. Therefore, in comparison with the Fe/MCM-41(2.5) sample, it is important to note that the Fe/MCM-RHA(2.5) sample presents a higher proportion of the active Fe^3+^ species. In addition, the higher catalytic efficiency of the MCM-RHA series could also be associated with the nature of adsorption. Thus, the degradation reaction of the pollutant could be favored on the surface of the material due to the aforementioned chemisorption process of the dye molecules on the surface of the MCM-RHA.

For instance, Table 5 summarizes a comparison between the two best Fe-containing catalysts supported on MCM-41 and MCM-RHA, and selected works from the literature that refer to RhB photodegradation are referenced.

It is clear that some works reported in Table 5 show that not only MCM-41-based catalysts, such as TiO_2_ and MnO_2_, presented high efficiency in the remediation of RhB. Values of remediation of up to ca. 99% and 100% for the removal and the degradation of RhB by adsorption and photocatalytic degradation, respectively, can be seen. Some of these results are higher than those observed in the best catalysts of the present work; however, it should be noted that the loading used in the present work is remarkably lower (0.1 g·L^−1^) compared to the other works [65,66,67]. In addition, we consider that a comparison of results between Fe_2_O_3_ catalysts and Fe/MCM catalysts is not fair, because the specific surface area of the MCM-based catalysts is much higher than that presented by Fe-based nanoparticles. However, a very interesting work on the Fe_2_O_3_/ZnO photocatalyst [68] has been included in Table 5. It can be seen that in spite of the fact that the surface area is remarkably lower than that reported in the Fe/MCM-41 and Fe/MCM-RHA catalysts of the present study, the photocatalytic activity of Fe_2_O_3_/ZnO achieved up to 95% RhB degradation. An explanation for this high photoactivity is that the catalysts’ loading was up to ca. 0.7 g·L^−1^, which is seven times higher than the loading used in the present work, as described in the Section 3.3.

**Figure 11:**
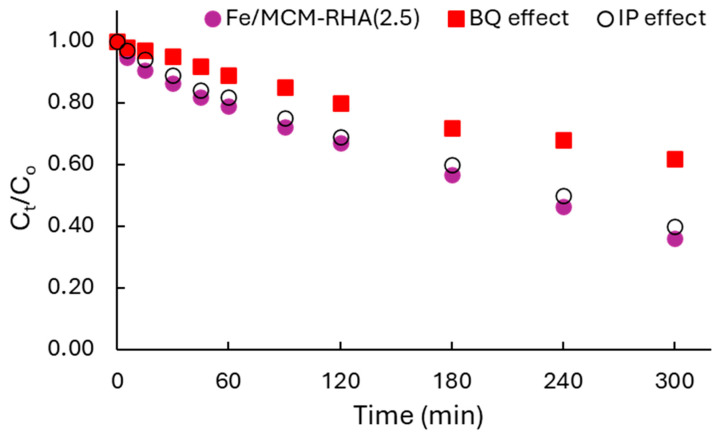
Scavenger study for Fe/MCM-RHA(2.5) catalyst.

Finally, in addition to the adsorption mechanism of RhB, discussed in detail in Section 2.2, another issue to be discussed concerns the mechanism of RhB photodegradation. This was verified by careful scavenger tests, verifying the influence of reactive oxygen species (ROS), mainly O_2_^•–^ and ^•^OH radicals, on the kinetics of RhB degradation. Section 3.4 described the methodology used for these tests. The Fe/MCM-RHA(2.5) catalyst was selected to perform this study because it was the best catalyst of the present work. Thus, Figure 11 shows the influence of the use of benzoquinone (BQ) and isopropyl alcohol (IP) on the activity of the catalysts.

It can be seen in Figure 11 that the photocatalytic activity of RhB in the presence of BQ as a scavenger was clearly suppressed while in the presence of IP, and the photoactivity was almost negligible affected. These results suggest that the mechanism of reaction is preferentially occurring by superoxide radicals (O_2_^•–^). This result suggests that the reaction mechanism proceeds via a unimolecular electrophilic addition (E1) pathway, as was also reported by our group [64] for the photodegradation of the azo-dye basic blue 41.

## 3. Materials and Methods

### 3.1. Synthesis of MCM-41, MCM-RHA, and Fe-Based Catalysts

An ordered mesoporous MCM-41 material was prepared according to previous works [39]. In a typical synthesis, cetyltrimethylammonium bromide (CTAB) from Merck (Rahway, NJ, USA, 99%) was used as a template and dissolved in distilled water at 40 °C (0.79 Mol/L). In addition, 60 mL sodium silicate extracted from rice husk was used as a biogenic silica precursor, which was titrated with sulfuric acid (H_2_SO_4_) aqueous solution 4 M, under constant stirring, until pH ca. 10.5. The resulting mixture was vigorously stirred for 6 h at room temperature and the gel obtained was thermally treated at 100 °C in autoclave reactors for 1 day. The solid was extensively washed with distilled water and dried at 60 °C overnight. Finally, the template agent was removed, the material was heated under N_2_ flow (2 °C/min and 5 mL/min) to 500 °C for 6 h, and then it underwent calcining at 500 °C for 6 h under air flow (2 °C/min and 5 mL/min). The so-prepared ordered mesoporous material was labelled as MCM-RHA. For the sake of comparison, ordered mesoporous MCM-41 was also synthesized according to the method reported by Cuello et al. [40]. The synthesis of MCM-41 was carried out under basic pH conditions, using cetyltrimethylammonium bromide (CTAB) and high-purity tetraethoxysilane (TEOS) from Merck (USA, 99%) as the template and silicon source, respectively. The obtained solid was filtered off, washed with distilled water, and dried at 60 °C overnight. Then, the template was removed under N_2_ flow to 500 °C and subsequently calcined at 500 °C under air flow.

MCM-41 and MCM-RHA were subsequently modified with Fe using the wetness impregnation method. Aqueous solutions of iron nitrate [Fe(NO_3_)_3_·9H_2_O, Merck USA, >95%] were prepared with a concentration corresponding to the nominal iron loadings with respect to MCM-41 and MCM-RHA. The solids were dried at 60 °C overnight and calcined at 350 °C for 3 h. The materials were identified as Fe/MCM-41(x) and Fe/MCM-RHA(x), where x symbolizes the nominal Fe content (2.5, 5, or 10% *w*/*w*).

### 3.2. Characterization

The materials were characterized following different methods previously reported [21,39,40,42]. N_2_ adsorption/desorption isotherms were obtained at –196 °C (N_2_, 99.99% purity) using an ASAP 2000 equipment (Micromeritics, USA). The samples were degassed before analysis at 300 °C for 6 h under vacuum. The specific surface (S_BET_) was determined using the Brunauer–Emmett–Teller method (BET). The total volume of pores (V_TP_) was estimated at a relative pressure of ca. 0.98. The pore size distributions of the samples were determined using the BJH (Barrett–Joyner–Halenda) method.

The crystalline framework of the samples was analyzed by X-ray diffraction (XRD) using a X-Pert Pro (PANalitic, Houston, TX, USA) diffractometer with CuKα radiation of 1.5418 Å. All diffraction data were recorded in the range of 2–7°, at an interval of 0.01° and with a scan rate of 0.6°·min^−1^. Inductively coupled plasma atomic emission spectroscopy (ICP-AES) was used to analyze the composition of elements presented in samples using a) ICP-OPTIMA 2100 DV spectrophotometer (Perkin Elmer, USA). The morphology was studied by scanning electron microscopy (SEM), using FE-SEM Σigma (20 kV) equipment. Gold coverage was applied to make samples conductive. Transmission electron microscopy (TEM) images were also obtained in a JEM-2100 Plus 200 kV (JEOL, Peabody, MA, USA). The diffuse reflectance UV–Visible (DR/UV–Vis) spectra were obtained on a V-650 spectrophotometer (Jasco, Tokyo, Japan) with an integrating sphere (Jasco International, Tokyo, Japan). XPS analyses were performed on a multi-technique Dual X-ray source Mg/Al model XR50 (Specs, Berlin, Germany) and a hemispherical analyzer (150 PHOIBOS) with a fixed transmission mode analyzer (FAT). The range of wavelengths used was 200–900 nm. Fourier transformed infrared (FTIR) spectral measurements of pyridine adsorption on the samples were also performed to evaluate the strength and type of acid sites. Spectra were recorded by a Nicolet iS10 (Thermo Scientific, Waltham, MA, USA) instrument in the scan range of 400–4000 cm^−1^. Supported pellets of the samples (ca. 20 mg and 13 mm diameter) were prepared and placed in a cell with a CaF_2_ window and evacuated for 7 h at constant temperature (400 °C) under a dynamic vacuum. The residual pressure was less than 10^−3^ Pa. The background spectrum was recorded after cooling the sample to room temperature. Afterwards, the solid pellet was exposed to pyridine vapors (Sintorgan, Villa Martelli, Argentina, 99% purity) until it saturated the system to 46 mm Hg at room temperature. This pressure remained for 12 h. After this time, an IR spectrum of the adsorbed pyridine is recorded. Subsequent IR spectra were obtained following the pyridine desorption by evacuation for 1 h at 50, 100, and 200 °C. The spectrum for each sample was obtained by subtracting the background previously recorded.

The electrochemical study of MCM-41, MCM-RHA, and Fe-based catalysts was carried out by a drop coating method of glassy carbon electrodes described as follows. First, 2 mg of solid to analyze is dispersed in 1 mL of 1 wt. % solution of sodium dodecyl sulfate (SDS), prepared in MilliQ water using a 1.5 mL Eppendorf tube. The dispersion is first mixed by manual shaking, and then the tube was submitted to ultrasound for 10 min. This step is repeated three times. Commercial glassy carbon electrodes (GCEs) were washed and polished with 0.3 and 0.05 µm particle-sized alumina. GCEs are rinsed with MilliQ water and allowed to dry at room temperature. GCEs were modified by drop coating on the surface with 5 μL of the solid dispersed in 1% SDS and then left to dry at room temperature for 30 min. To improve the stability of the samples on the electrode surface, 5 μL of 2.5% Nafion solution (prepared from a 5 wt.% solution by dilution with absolute ethanol) was added. The so-stabilized electrode was left to dry again at room temperature for 15 min or until dryness was reached. The electrochemical measurements were performed in a conventional 3-electrode cell using a 0.1 M phosphate-buffered saline (PBS) as the electrolyte solution, with a constant pH of ca. 6.8. The working electrodes (modified GCEs) are immersed in the solution for 10 min prior to electrochemical measurement, and a Pt wire and 1M Ag/AgCl electrode were used as counter and reference electrodes, respectively. Cyclic voltammetry was performed from –0.2 V to +0.7 V, with a positive initial sense in each measurement, at a sweep rate of 50 mV·s^−1^.

The surface pH (pH_PZC_) of catalysts was measured using the pH-drift procedure [21]. In a typical test, 20 mg catalyst was suspended into 20 mL water (grade MilliQ) for a mass proportion of 0.1 wt.%. The pH was followed under stirring until constant pH was reached, using a pH meter ORP model 8601D (Mettler Toledo, Columbus, OH, USA). The pH of the suspension in the steady-state condition corresponds to the pH at the point-zero charge of the surface (pH_PZC_).

### 3.3. Kinetics of Adsorption and Photodegradation of RhB

High-purity rhodamine B (RhB) from Sigma-Aldrich was used for the adsorption and photodegradation tests. Kinetics studies of adsorption in the dark were performed using a RhB solution (250 mL) with an initial concentration of ca. 5.5 mg·L^−1^ (ca. 11.5 μmol·L^−1^, 2.875 μmol), mixed with the catalyst (25 mg), and the kinetics of adsorption were followed for 120 min. The equilibrium condition was typically achieved within 45–60 min for most of the catalysts. Accordingly, before starting the irradiation of catalysts, a preliminary period of 60 min of RhB adsorption in the dark was performed. The pseudo first-order, the pseudo-second-order, and the kinetic constants according to the intraparticle diffusion model were estimated from the kinetic data in the range of 0–60 min adsorption.

For the photocatalytic tests, a 350 mL Pyrex photochemical reaction vessel was used, with UV irradiation provided by a high-pressure Hg lamp of 450 W, emitting 250 W·m^−2^ at 360 nm (Sigma-Aldrich, USA). Figure S8 (SI) shows the emission spectra of the Hg lamp. The lamp was vertically suspended inside a cylindrical double-walled quartz jacket cooled at 10 °C by a circulating water flow system immersed in the solution. Each test involved 250 mL of RhB solution and 25 mg of catalyst, maintaining a constant catalyst loading of ca. 0.1 g·L^−1^. The RhB concentration was estimated from UV-Vis spectra at 554 nm using a UV/Vis spectrophotometer (Spectroquant^®^ Prove 300, Merck). The direct photolysis was verified as a blank test, and TiO_2_-P25 (Evonik, Essen, Germany) was used as the benchmark photocatalyst. All experiments were performed at least in duplicate, with an experimental reproducibility higher than 95%.

### 3.4. Scavenger Tests for Identification of Active Species ^•^OH and O_2_^•–^

We assessed the active species ^•^OH and O_2_^•–^ generated during photocatalysis through scavenger tests [64]. As reported elsewhere [67], benzoquinone (BQ) and isopropanol (IP) were used as scavengers for O_2_^•–^ and ^•^OH radicals, respectively. In a typical procedure, ca. 14 μmol·L^–1^ of BQ was added to 0.250 L of RhB solution (5.5 mg·L^−1^) for identifying superoxide radicals (O_2•–_). The same procedure was used for identifying hydroxyl radicals (_•_OH), where ca. 0.9 mol·L^–1^ of IP was employed.

## 4. Conclusions

MCM-41 and MCM-RHA mesoporous silicates were successfully synthesized using traditional silica precursors and rice husk as natural silica precursors, respectively; then they were modified with different amounts of Fe using the wet impregnation method. This alternative method of synthesis proved to be effective, simple, inexpensive, and environmentally friendly.

All materials showed a high specific surface, pore volume, and good structural regularity, indicating that the structure was preserved after metal incorporation. The well-ordered mesoporous structure of the materials was confirmed by TEM images, and the iron species distribution was inferred from the ICP and UV–Vis/DR analysis.

From the electrochemical characterization, it can be concluded that modifications of GCE with MCM compounds decrease the electroactive area of the studied electrode surfaces. In addition, contrary to what was expected, the Fe species embedded in the silica-based materials did not reflect a redox behavior, which was probably because of an important confining effect of most of the nanoparticles within the porous framework of MCM materials.

It can be concluded that MCM-like materials can be prepared by using a natural and renewable Si source, obtaining ordered mesoporous structures like those already prepared using a high-cost commercial organic silica (tetraethoxysilane). These mesostructured materials were successfully tested in the photocatalytic degradation of rhodamine B in water. The results indicated that the catalyst with the lowest iron loading (2.5% *w*/*w* Fe) achieved the highest degradation of contaminants, likely due to the presence of active Fe species that are highly dispersed and interact effectively with the support surface.

## Figures and Tables

**Table 1:** Summary of properties of Fe-containing MCM-41 and MCM-RHA catalysts.

Samples	Fe Content (wt. %) ^a^	S_BET_ (m^2^·g^−1^) ^b^	V_TP_ (cm^3^·g^−1^) ^c^	D_p_ (nm) ^c^	pH_PZC_ ^d^
MCM-41	-	996	0.70	3.5	6.14
Fe/MCM-41(2.5)	2.6	948	0.71	3.5	6.08
Fe/MCM-41(5)	4.5	923	0.69	3.5	5.95
Fe/MCM-41(10)	8.4	801	0.59	3.5	5.73
MCM-RHA	-	1022	0.91	3.0	5.89
Fe/MCM-RHA(2.5)	2.3	889	0.74	2.6	5.74
Fe/MCM-RHA(5)	4.7	877	0.66	2.8	5.59
Fe/MCM-RHA(10)	8.7	761	0.62	2.6	5.29

^a^ Obtained by ICP-AES analysis. ^b^ S_BET_ is the specific surface area obtained by BET equation. ^c^ V_TP_ is the total volume and D_p_ is the average diameter of pores determined using the BJH method. ^d^ pH_PZC_ is the pH of the solid at the point-zero charge obtained by the drift method [21].

**Table 2:** Summary of electrochemical parameters obtained at a scan rate of 0.05 V·s^−1^.

Electrode	Voltammogram Area (μA·V^−1^)	Electric Charge (μC)
GCE	0.921	18.42
GCE-SDS-Nafion	0.797	15.94
GCE-MCM-41	0.788	15.76
GCE-Fe/MCM-41(2.5)	0.720	14.40
GCE-Fe/MCM-41(5)	0.739	14.78
GCE-Fe/MCM-41(10)	0.805	16.10
GCE-MCM-RHA	0.755	15.10
GCE-Fe/MCM-RHA(2.5)	0.700	14.00
GCE-Fe/MCM-RHA(5)	0.444	8.88
GCE-Fe/MCM-RHA(10)	0.621	12.42
GCE-Fe_2_O_3_	0.607	12.14

**Table 3:** Summary of the kinetic parameters obtained for the rhodamine B (RhB) adsorption in the dark.

Samples	q_eq_^a^ (μmol)	k_1_^b^ (min^−1^)	R^2^_k1_^c^	k_2_^d^ (μmol^−1^·min^−1^)	R^2^_k2_^e^	k_IPD_ ^f^ (μmol·min^−1/2^)	R^2^_IPD_^g^	C_IPD_ ^h^ (μmol)
TiO_2_-P25	0.22	0.026	0.912	0.380	0.988	0.018	0.895	0.080
MCM-41	2.35	0.036	0.987	0.066	0.951	0.204	0.948	0.732
Fe/MCM-41(2.5)	1.96	0.041	0.986	0.076	0.935	0.184	0.941	0.506
Fe/MCM-41(5)	1.79	0.031	0.980	0.059	0.937	0.151	0.976	0.564
Fe/MCM-41(10)	1.69	0.030	0.976	0.064	0.942	0.137	0.979	0.582
MCM-RHA	1.91	0.032	0.960	0.064	0.995	0.153	0.918	0.726
Fe/MCM-RHA(2.5)	1.91	0.042	0.983	0.108	0.945	0.152	0.915	0.718
Fe/MCM-RHA(5)	1.68	0.034	0.984	0.071	0.980	0.163	0.927	0.412
Fe/MCM-RHA(10)	1.60	0.037	0.963	0.074	0.972	0.122	0.963	0.656

^a^ q_eq_ is the rhodamine B (RhB) adsorbed at equilibrium condition after 60 min. ^b^ k_1_ is the pseudo-first-order rate-constant. ^c^ R^2^_k1_ is the quadratic linear factor for the first-order kinetic constant. ^d^ k_2_ is the pseudo-second-order rate-constant. ^e^ R^2^_k2_ is the quadratic linear factor for the second-order kinetic constant. ^f^ k_IPD_ is the rate-constant according to IPD model. ^g^ R^2^_IPD_ is the quadratic linear factor for the IPD kinetic rate-constant. ^h^ C_IPD_ is the boundary layer thickness constant for the IPD model.

**Table 4:** Summary of kinetic parameters obtained for the RhB photocatalytic degradation.

Samples	k_app_^a^ × 10^−3^ (min^−1^)	R^2^_kapp_^b^	k_app-i_/k_lysis_^c^	k_app-i_/k_TiO2_^d^	C_5h_^e^ (%)	v_sur_^f^ (μmol.min^−1^)
Photolysis	1.6	0.94	1.0	0.5	20	0
TiO_2_-P25	3.4	0.98	2.1	1.0	58	0.00075
MCM-41	4.3	0.97	2.7	1.3	51	0.01011
Fe/MCM-41(2.5)	7.5	0.98	4.7	2.2	60	0.01470
Fe/MCM-41(5)	4.4	0.96	2.8	1.3	55	0.00788
Fe/MCM-41(10)	3.2	0.89	2.0	0.9	46	0.00541
MCM-RHA	2.7	0.97	1.7	0.8	47	0.00516
Fe/MCM-RHA(2.5)	7.4	0.97	4.6	2.2	74	0.01413
Fe/MCM-RHA(5)	3.7	0.96	2.3	1.1	57	0.00622
Fe/MCM-RHA(10)	2.4	0.99	1.5	0.7	29	0.00384

^a^ k_app_ is the first-order apparent rate-constant for the RhB photodegradation obtained from the plot of Equation (4). ^b^ R^2^_kapp_ is the quadratic regression factor for k_app_. ^c^ k_app-i_/k_lysis_ is the photoactivity relative to direct photolysis. ^d^ k_app-i_/k_TiO2_ is the photoactivity relative to TiO_2_. ^e^ C_5h_ is the RhB photoconversion after 5 h of UV irradiation. ^f^ v_sur_ is the global surface reaction estimated using Equation (5).

**Table 5:** Comparison of photocatalytic activity of the present materials and selected works from the literature in the RhB photocatalytic degradation.

Materials	Surface Area (m^2^·g ^−1^)	Pore Diameter (nm)	Pore Volume (cm^3^·g ^−1^)	Adsorption (%)	Degradation (%)	Reference
Fe/MCM-41(2.5)	948	3.5	0.71	68	60	Present work
Fe/MCM-RHA(2.5)	889	2.6	0.74	66	74	Present work
Ti-MCM-41-10	1219	2.0	0.82	90–96	90	[65]
MnO_2_-MCM-41	1313	2.1	0.86	99	100	[66]
TiO_2_/MCM-41	620–724	4.2	Not reported	62–75	75–78	[67]
Fe_2_O_3_/ZnO	34–44	2.2–14.2	Not reported	Not reported	95	[68]

## Data Availability

Additional data and materials can be made available on request.

## Supplementary Materials

The following supporting information can be downloaded at: https://www.mdpi.com/article/10.3390/molecules30122484/s1, Table S1: Kinetic models used for the analysis of RhB adsorption on catalysts; Figure S1: SEM-EDS analysis for Fe/MCM-RHA(10) catalyst; Figure S2: UV-visible spectra for Fe-based catalysts; Figure S3: XPS spectra in the Fe 2p core level for Fe/MCM-RHA(10) catalyst; Figure S4: Adsorption of RhB on Fe-based catalysts supported on MCM-based materials in terms of different kinetic models. (a–c): Fe/MCM-41(2.5). (d–f): Fe/MCM-RHA(2.5). (a,d): First-order kinetics. (b,e): Second-order kinetics. (c,f): Intraparticle diffusion (IPD) kinetic model; Figure S5: Adsorption of RhB on Fe-based catalysts supported on MCM-based materials in terms of different kinetic models. (a-c): Fe/MCM-41(5). (d-f): Fe/MCM-RHA(5). (a,d): First-order kinetics. (b,e): Second-order kinetics. (c,f): Intraparticle diffusion (IPD) kinetic model; Figure S6: Adsorption of RhB on Fe-based catalysts supported on MCM-based materials in terms of different kinetic models. (a–c): Fe/MCM-41(10). (d–f): Fe/MCM-RHA(10). (a,d): First-order kinetics. (b,e): Second-order kinetics. (c,f): Intraparticle diffusion (IPD) kinetic model; Figure S7: Adsorption of RhB on TiO_2_-P25 in terms of different kinetic models. (a): First-order kinetics. (b): Second-order kinetics. (c): Intraparticle diffusion (IPD) kinetic model; Figure S8: Emission spectra of Hg lamp.
