# Supplementary material for: Rice Husks as a Biogenic Template for the Synthesis of Fe2O3/MCM-41 Nanomaterials for Polluted Water Remediation
Source: Molecules. 2025 Jun 6;30(12):2484. doi: 10.3390/molecules30122484 (PMC12195685; doi:10.3390/molecules30122484)
Supplement: Supplementary file 1 [file molecules-30-02484-s001.zip › molecules-3657114-supplementary.pdf]

Supplementary Information

Rice-husks as a biogenic template for the synthesis of Fe-containing MCM-41 nanomaterials for polluted water remediation

Tamara B. Benzaquén [1,*], Paola M. Carraro [1], Griselda A. Eimer [1], Julio Urzúa-Ahumada [2], Po S. Poon [3], Juan Matos [4,*]

[1]Centro de Investigación y Tecnología Química (CITeQ), UTN-CONICET, Maestro Marcelo López esq. Cruz Roja Argentina, 5016ZAA Córdoba, Argentina.

[2]Proyecto ANILLO: Efficient use of Water for the Sustainable Agriculture under Climate Change Condition (H_2O-SAC[3]). Universidad Autónoma de Chile, 8900000 Santiago, Chile.

[3]Unidad de Desarrollo Tecnológico (UDT), Universidad of Concepción, Barrio Universitario s/n, Concepción, Chile.

[4]Unidad de Cambio Climático y Medio Ambiente (UCCMA), Instituto Iberoamericano de Desarrollo Sostenible (IIDS), Facultad de Arquitectura, Construcción y Medio Ambiente, Universidad Autónoma de Chile, Temuco 4780000, Chile.

* Corresponding authors.

Emails: juan.matos@uautonoma.cl (J. Matos); tbenzaquen@gmail.com (T.B. Benzaquen).

Phone: +56 9 93798340

Table S1. Kinetic models used for the analysis of RhB adsorption on catalysts.

Kinetic model	Equation	Reference
**Pseudo-first-order**	$\log(q_{eq} - q_t) = \log(q_t) - (k_1/2.303)\ t$	[22,59-62]
**Pseudo-second-order**	$[1/(q_{eq} - q_t)] = (1/q_{eq}) + k_2\ t$	[22,60]
**Intraparticle diffusion**	$q_t = C_{IPD} + k_{IPD}\ t^{1/2}$	[22,60-63]

The pseudo-first-order model showed in Eq. (S1) where k_1 is the pseudo-first-order rate constant (min^{-1}) for the adsorption, q_t is the amount of RhB adsorbed (in μmol), at time t (min), and q_{eq} is the amount of RhB adsorbed at equilibrium (in μmol).

$$dq_t/dt = k_1(q_{eq} - q_t) \quad \text{(S1)}$$

The integration of Eq. (S1) at the initial conditions ($q_t = 0$ at $t = 0$) yields the Eq. (S2):

$$\log(q_{eq} - q_t) = \log(q_{eq}) - (k_1/2.303).t \quad \text{(S2)}$$

In addition, a pseudo-second-order equation may be expressed by Eq. (S3):

$$dq_t/dt = k_2(q_{eq} - q_t)^2 \quad \text{(S3)}$$

Where k_2 is the pseudo-second-order rate constant ($\mu mol^{-1}.min^{-1}$) for the adsorption.

Applying the initial conditions, Eq. (S3) can be integrated to obtain:

$$[1/(q_{eq} - q_t)] = (1/q_{eq}) + k_2.t \quad \text{(S4)}$$

The impact of intraparticle diffusion on the adsorption capacity of RhB was examined, considering the fractional approach to equilibrium, which is a function of $(D_t/r^2)^{1/2}$, where r is the radius of the adsorbent particle and D is the effective diffusivity of the solute within the particle. The initial rate according to the intraparticle diffusion model (IPD) is obtained from the linear regression of the plot $q_t = f(t^{1/2})$, expressed by the Eq. (S5), where k_{IPD} is the IPD rate-constant expressed in $\mu mol.min^{-1/2}$, and C_{IPD} is the IPD capacity constant (μmol) attributed to the extension of the boundary layer thickness.

$$q_t = C_{IPD} + k_{IPD}.t^{1/2} \quad \text{(S5)}$$

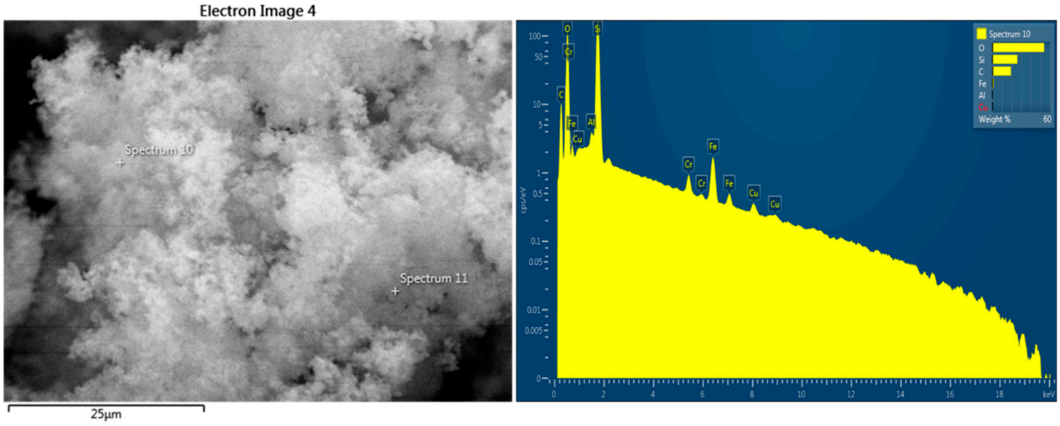

Element	Line Type	Apparent Concentration	k Ratio	Wt%	Wt% Sigma	Standard Label	Factory Standard
C	K series	4.82	0.04823	19.10	0.20	C Vit	Yes
O	K series	85.10	0.28638	53.67	0.16	SiO2	Yes
Al	K series	0.26	0.00187	0.16	0.01	Al2O3	Yes
Si	K series	43.99	0.34860	25.78	0.09	SiO2	Yes
Fe	K series	1.65	0.01650	1.13	0.03	Fe	Yes
Cu	K series	0.22	0.00219	0.16	0.03	Cu	Yes
Total:				100.00			

Figure S1. SEM-EDS analysis for Fe/MCM-RHA(10) catalyst.

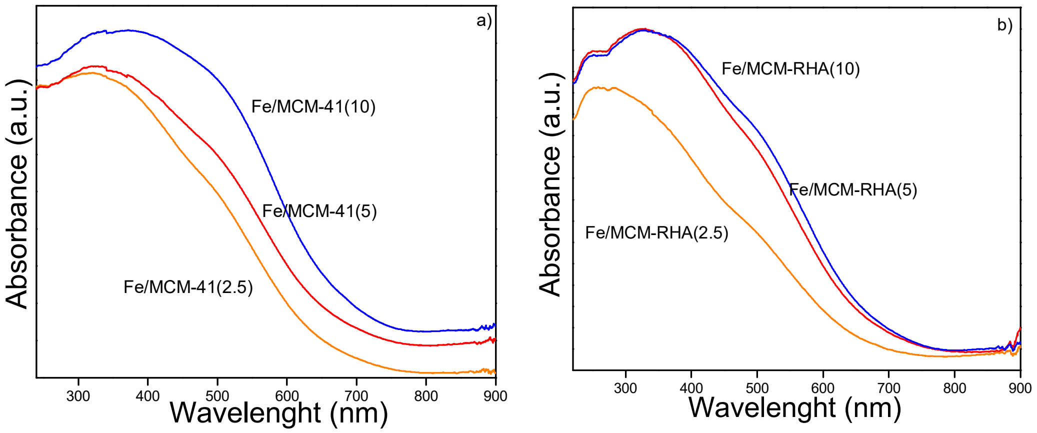

Figure S2. UV-visible spectra for Fe-based catalysts.

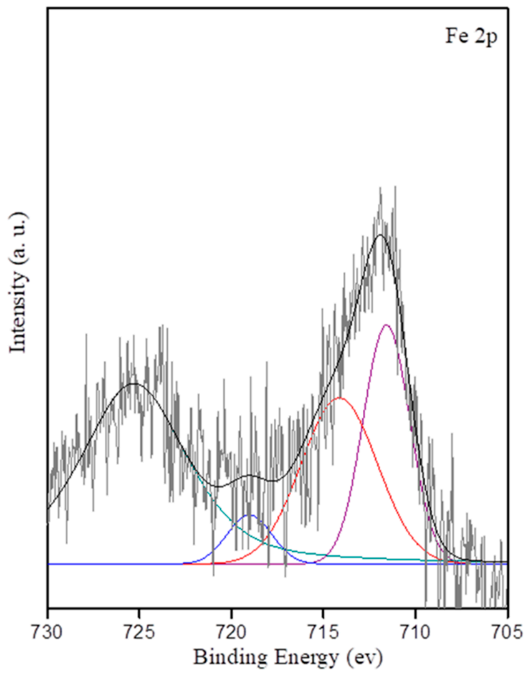

Figure S3. XPS spectra in the Fe 2p core level for Fe/MCM-RHA(10) catalyst.

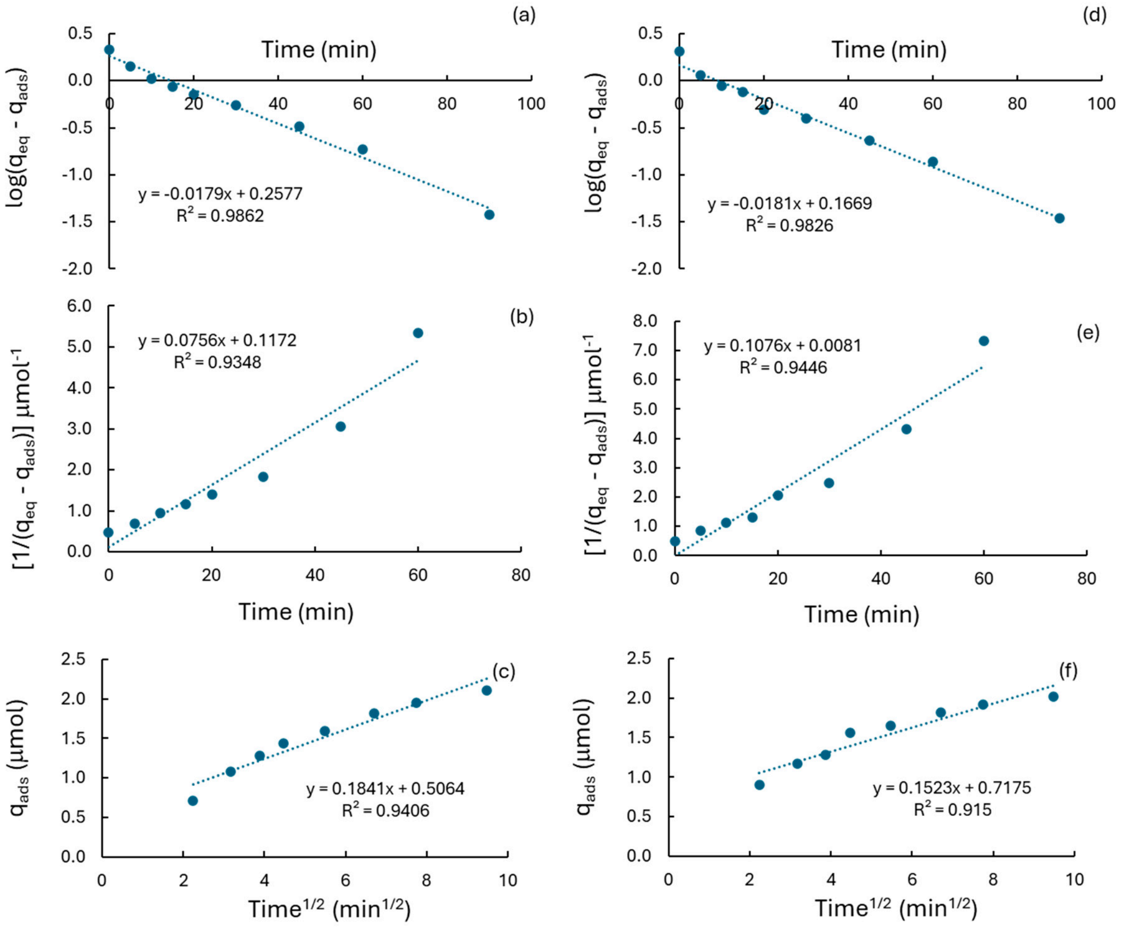

Figure S4. Adsorption of RhB on Fe-based catalysts supported on MCM-based materials in terms of different kinetic models. (a-c): Fe/MCM-41(2.5). (d-f): Fe/MCM-RHA(2.5). (a,d): First-order kinetics. (b,e): Second-order kinetics. (c,f): Intraparticle diffusion (IPD) kinetic model.

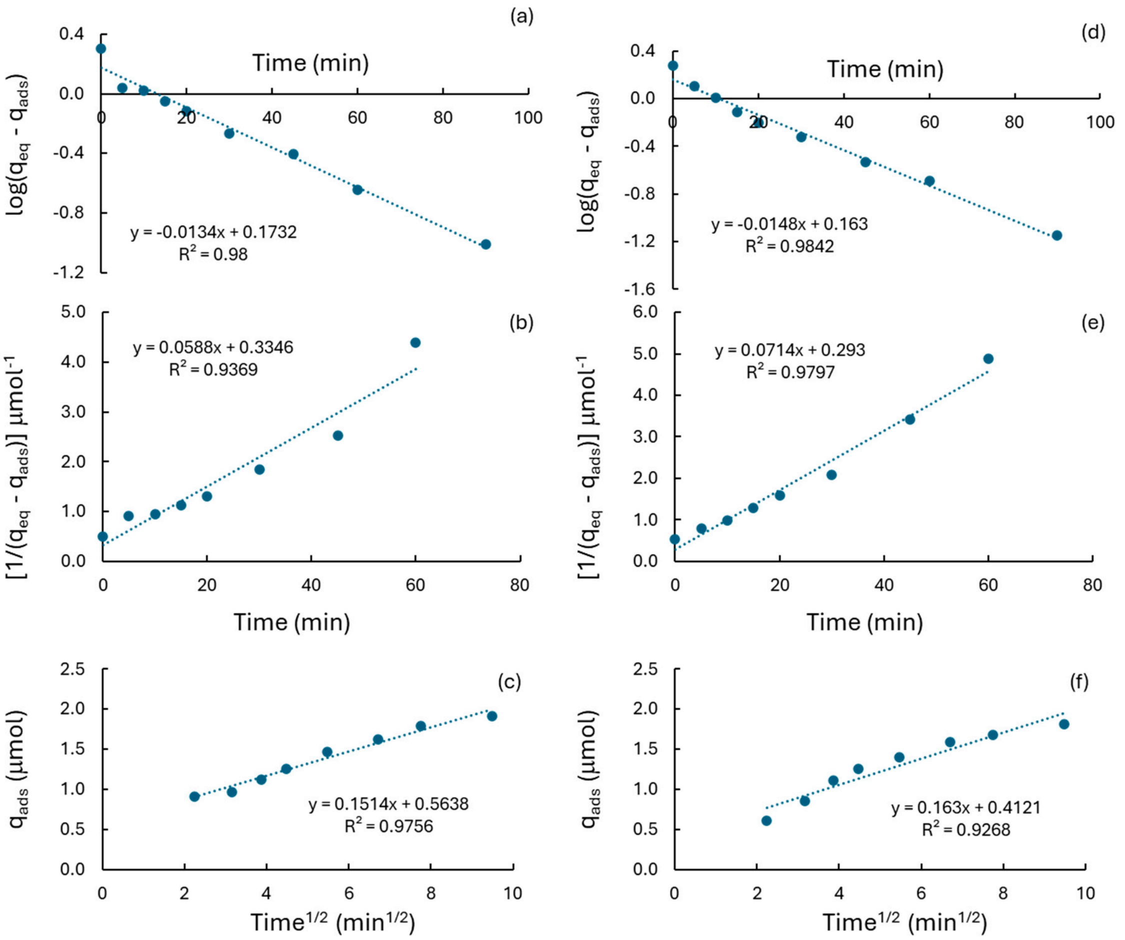

Figure S5. Adsorption of RhB on Fe-based catalysts supported on MCM-based materials in terms of different kinetic models. (a-c): Fe/MCM-41(5). (d-f): Fe/MCM-RHA(5). (a,d): First-order kinetics. (b,e): Second-order kinetics. (c,f): Intraparticle diffusion (IPD) kinetic model.

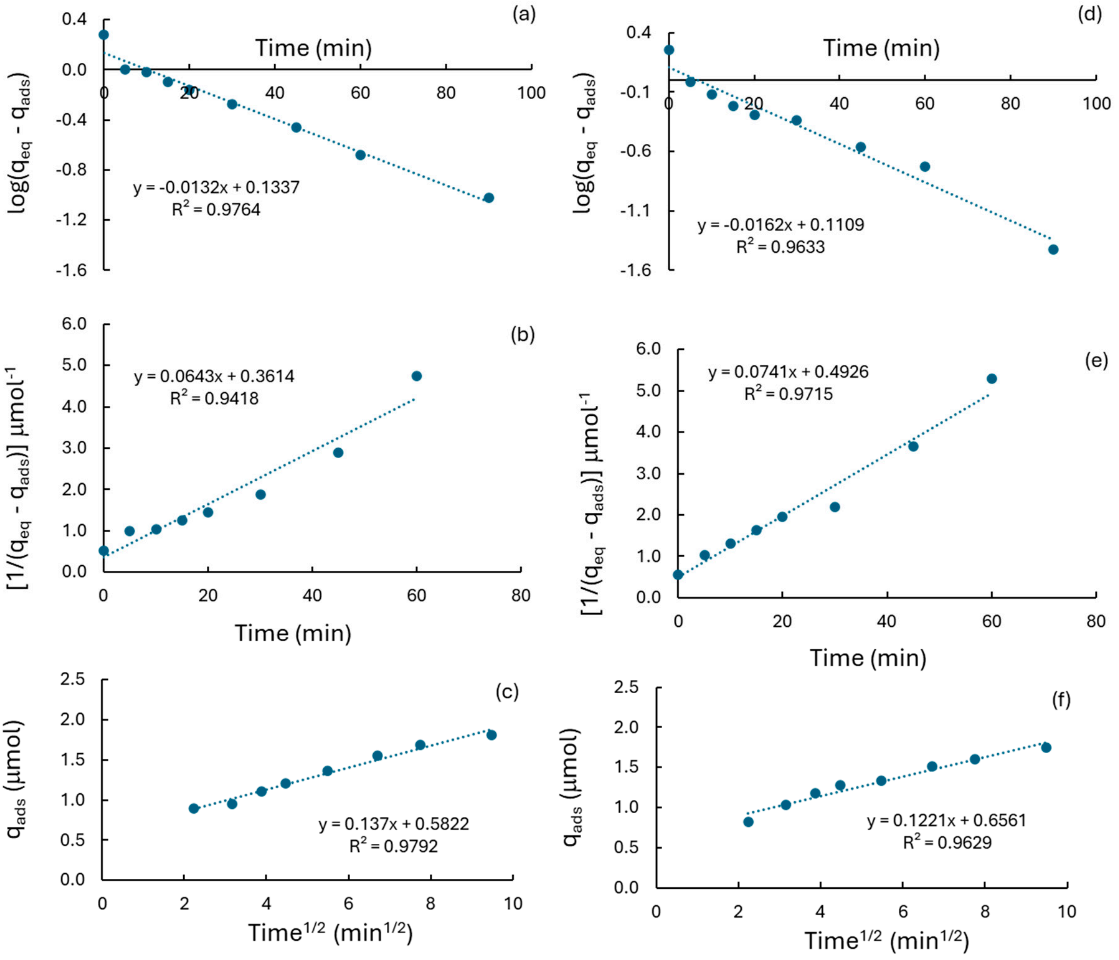

Figure S6. Adsorption of RhB on Fe-based catalysts supported on MCM-based materials in terms of different kinetic models. (a-c): Fe/MCM-41(10). (d-f): Fe/MCM-RHA(10). (a,d): First-order kinetics. (b,e): Second-order kinetics. (c,f): Intraparticle diffusion (IPD) kinetic model.

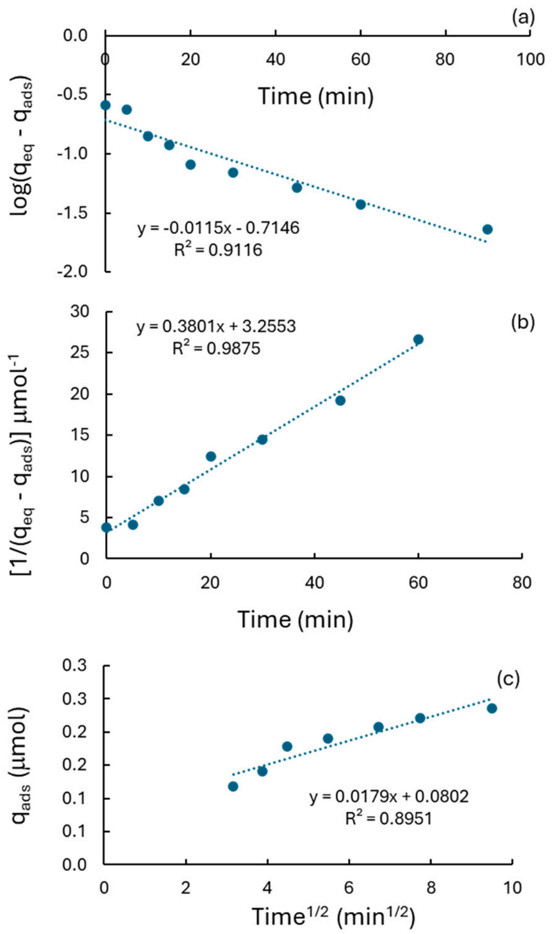

Figure S7. Adsorption of RhB on TiO_2-P25 in terms of different kinetic models. (a): First-order kinetics. (b): Second-order kinetics. (c): Intraparticle diffusion (IPD) kinetic model.

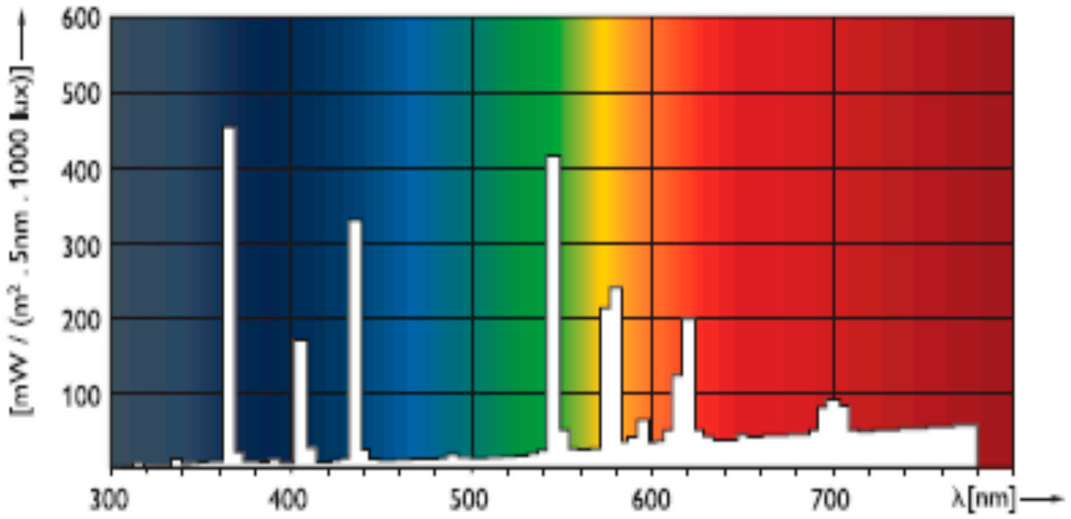

Figure S8. Emission spectra of Hg lamp.
